# Reduced Serially Improved GTOs for Molecular Applications from H to Ar: Efficient Diffuse Functions in Augmented Basis Sets

**DOI:** 10.1021/acs.jpca.5c05079

**Published:** 2025-11-24

**Authors:** Ignacio Ema, Jesús San-Fabián, Guillermo Ramírez, Rafael López, José Manuel García de la Vega

**Affiliations:** Departamento de Química Física Aplicada, 16722 Universidad Autónoma de Madrid, Madrid E-28049, Spain

## Abstract

A new family of Serially Improved Gaussian-type Orbitals for Molecular Applications (SIGMA) has been developed for hydrogen to argon and is available in both standard and augmented versions. This new basis set features a reduced number of primitive functions compared to the original SIGMA set and eliminates the constraint of shared exponents across different angular momentum shells. Its composition is similar to that of the Dunning correlation-consistent basis sets. In the augmented version of this novel SIGMA family, termed reduced SIGMA, the diffuse primitive functions are contracted with others bearing higher exponents, unlike in the corresponding Dunning sets, where they are added to the set without contraction. This feature makes the reduced SIGMA basis sets less susceptible to linear dependencies in large and compact systems, which is reflected in better convergence of the minimization process and has a positive impact on energy optimization procedures. The improved performance of the augmented reduced SIGMA basis sets over Dunning and other commonly used basis sets is demonstrated through atomic and molecular calculations at various computational levels across a broad set of systems.

## Graphical abstract

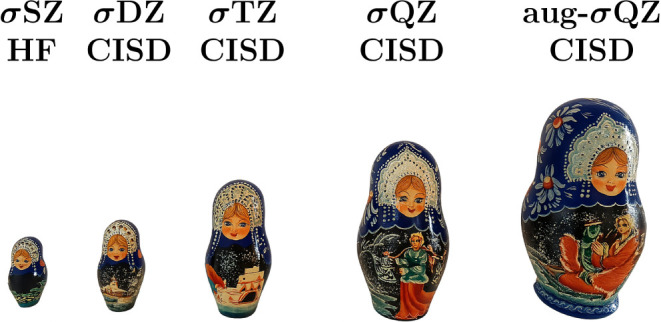

## 1 Introduction

Electronic structure calculations have become powerful tools in many areas of chemistry, including the design of new materials, drug discovery, chemical reactions, the study of substance properties, and molecular interactions. Today, quantum chemistry methods can handle large molecules with reasonable accuracy and at an affordable computational cost. Most molecular calculations rely on wave functions constructed from some type of one-electron functions—namely, orbitals (or spin-orbitals if the spin component is included). These functions are typically expressed as linear combinations of a set of functions, the basis set (BS), and consist of atom-centered products of exponentially decaying radial functions multiplied by angular functions, which are usually represented by either spherical harmonics or powers of Cartesian coordinates.

In molecular calculations, a common choice for the radial part is simple exponentials of the form $e^{-\alpha r^{2}}$ , where α represents suitable parameters and *r* is the distance to the nucleus of the atom to which the function belongs. The BS functions constructed in this way are called Gaussian-type orbitals (GTOs). The set of atomic orbital basis functions applied to a molecular system is one of the factors that most strongly influences the trade-off between speed and accuracy, and alongside the computational method, the BS determines the quality of the results. An important feature of the BS is its degree of completeness—that is, the quality of the Hilbert space it spans, reflected in its ability to provide a chemically meaningful electron wave function. In this regard, the description of valence electrons is crucial, as chemical behavior is largely determined by the outer electron cloud. Accordingly, BS are often classified using the so-called cardinal number, which indicates the number of independent basis functions per occupied valence orbital. The corresponding sizes are often referred to as double- (DZ), triple- (TZ), quadruple- (QZ) zeta, and so on, where the term zeta corresponds to the Greek symbol ζ, traditionally used to denote the parameters in the exponential part, and the prefix indicates the number of independent atomic radial functions per occupied valence orbital in the atomic ground state.

The minimal BS is the set that includes only the orbitals occupied by electrons. In actual calculations, it is generally advisable to avoid minimal BS, as their lack of flexibility can lead to uncontrollable errors. To enhance the versatility of the BS, additional polarization functions with higher angular momentum or diffuse functions with small exponents can be employed. This approach is essential for describing certain molecular systems and properties. To improve the performance, modern BS employ *contracted* GTO,^1^ which consist of linear combinations of the simple functions previously described, which are known as *primitive* functions.

As BS is a critical element in achieving optimal outcomes, significant effort has been devoted to its development since the early days of computational chemistry. This has led to the continuous enhancement of BS with new features, ensuring its ongoing relevance and effectiveness in the field.^2–11^ Thus, research into BS optimization continues to produce BS that enables fast and accurate chemical calculations scalable to larger systems than ever before. Intensive work has been carried out to devise basis sets and extrapolation schemes that allow us to approach the one-electron complete BS (CBS) limit as closely as possible, significantly impacting the development of chemical models.^9,12^

In the design of BS, special care must be taken in the case of systems where the diffuse part of the electron cloud plays a crucial role, as occurs when dealing with anions, atoms in different molecular environments, varying bond orders, highly polar systems, excited states, or noncovalent long-range interactions. In such cases, the usual standard BS are enlarged by including diffuse functions in the set, giving rise to the augmented versions of the BS, which provide sufficient flexibility for an accurate description of these systems.^13–15^ In spite of all this effort, there is one aspect that has hardly been addressed in the development of BS with diffuse functions—namely, the risk of overcompleteness in the BS when moving from atoms to molecules,^16^ particularly in geometrically compact systems. This overcompleteness arises mostly in the augmented BS when primitive functions with very small exponents are included directly, without contraction, and is reflected in the presence of a significant number of very small overlap eigenvalues, which indicate near-linear dependencies in the BS. This leads to reduced effectiveness in the minimization procedure and can sometimes result in poor convergence, a loss of accuracy, or convergence to an incorrect result. Furthermore, the presence of very small exponents in augmented BS has been linked to the lack of sparsity in the one-particle density matrix in large systems, which is a major source of difficulty for low-scaling approaches when using these BS.^15^

In this work, we present a new family of Serially Improved Gaussian-type Orbitals for Molecular Applications (SIGMA) BS (abbreviated as σBS) for elements from hydrogen to argon,^17^ offering both standard and augmented versions spanning double- to quadruple-ζ (DZ to QZ) quality. The σBS were initially developed for high-precision calculations^18–22^ with both their composition and development methodology differing fundamentally from those of conventional BS. The primary distinctions between this new family, referred to as reduced SIGMA BS (specifically σBS0), and the original σBS are that the exponents of the primitive functions in σBS0 are not shared between shells of different angular momentum (*L*) values, and the total number of primitive functions is reduced. In this regard, the σBS0 architecture more closely aligns with the established Dunning correlation-consistent BS,^23^ which serve as a benchmark in molecular computations, while demonstrating comparable performance in their standard form and superior performance in the augmented version (*a*σBS0).

Notably, *a*σBS0 exhibit enhanced convergence properties compared to the Dunning aug-cc-pVXZ^24^ counterparts. This improvement stems from two key features: the inclusion of higher exponents in the diffuse primitives and the systematic contraction of diffuse functions in the *s* and *p* shells with primitive functions having larger exponents. This design strategy effectively balances completeness and compactness, avoiding overcompleteness in dense systems while maintaining high accuracy. Thus, from a practical point of view, in comparison with other commonly used BS, the reduced SIGMA exhibit consistent convergence behavior in all cases, delivering results that outperform the most widely used BS both in terms of variational quality and geometry optimization.

The work is organized as follows. In the next section, the development of the σBS0 set is outlined. This is followed by a comparison of the performance of these BS with that of other commonly used sets of similar composition, taking the cc-pVXZ Dunning set as the primary reference and applying various computational methods to molecular systems of different sizes. Finally, the main conclusions drawn from this study are presented.

## 2 Basis Set Design

### 2.1 General Procedure

The new σBS0 proposed for the first three rows of the periodic table, from H to Ar, follow a stepwise construction scheme similar to that of the original SIGMA BS (matryoshka doll scheme) previously described^20,22^ and are closely related to the correlation-consistent BS family developed by Dunning et al.^23–32^ They are constructed in shells following Dunning’s scheme, in terms of the size of the contracted functions and their successive extensions. A key difference, however, is that at each step of the optimization procedure, only the newly added contracted functions are optimized, while those obtained in previous steps remain unchanged. For the initial set of contracted functions, the Hartree–Fock (HF) energy of the atomic ground state is used as the optimization criterion. Subsequent sets of contracted functions are optimized using the configuration interaction with single and double excitations (CISD). In atoms with multiple low-lying electronic configurations, the basis functions are optimized by considering all relevant configurations simultaneously. For alkali atoms, optimization is performed using the corresponding diatomic molecules instead of atomic excited states.

In summary, at each step of the procedure, the exponents from previous steps are kept fixed, while new primitive functions and a new shell are added and their exponents are optimized. The process begins with a minimal basis set optimized at the HF level, and each optimization step involves a simplex algorithm, followed by refinement through unidirectional scans for the nonlinear parameters (i.e., primitive exponents). Contraction coefficients are optimized using analytical rotations, maintaining the orthogonality of the basis functions. No gradients are used in this process. The optimization stops when a convergence of 10^–6^ is reached in the target function.

When extending the basis sets to their augmented versions, the anion is used for the optimization—except for alkaline earth metals and noble gases, where the anion is replaced by the diatomic molecule at its equilibrium distance due to the instability of the corresponding anions. In this regard, it should be noted that the inclusion of the anion in the BS optimization is not intended to achieve an accurate description of the anion itself but rather to enhance the description of the atom across a variety of molecular electron environments. As a consequence, it is not surprising that, despite the better variational performance of the *a*σBS0 over the corresponding Dunning aug-cc-pVXZ, the electron affinity values provided by the latter are slightly better than those of the former, which is due to the greater improvement achieved in the aσXZ for the ground state of the neutral atom than for the anion. Our current investigations are limited to sets of DZ, TZ, and QZ quality.

The following notation is used here: XZ refers to the Dunning cc-pVXZ basis sets, and aXZ refers to the aug-cc-pVXZ sets, with X = D, T, Q, etc. Accordingly, the new reduced SIGMA BS are designated σXZ0 for the standard versions and aσXZ0 for the augmented ones. The trailing zero distinguishes these reduced SIGMA sets from the existing σXZ and aσXZ versions, indicating that the polarization functions consist solely of primitive functions (without contraction), unlike in the original σBS,^20,22^ where each contracted polarization function includes two additional primitive functions—corresponding to σBS2 in this notation. Notice that the *s* and *p* shells of σBS0 consist of general contractions, as in the σBS.

With this new design affecting both the number of primitive functions and their exponents, the σXZ0 and aσXZ0 sets resemble the XZ and aXZ Dunning BS much more closely than the previous σXZ and aσXZ sets, enabling more direct comparisons of results. Upon analyzing the structure and performance of the (a)­σBS0, the set of primitive functions was slightly modified with respect to that of the (a)­XZ, as described in the next subsection. The purpose of these changes was to improve the description of the HF energy. The *a*σBS0 perform better than aXZ because the simultaneous reoptimization of all exponents is allowed when transitioning from σBS0 to *a*σBS0. This contrasts with the aXZ sets, in which the exponents of the primitive functions in XZ are kept unchanged when diffuse functions are added to describe the anion.

The presence of diffuse primitive functions with higher exponents in aσXZ0 than in the corresponding aXZ, and the fact that these diffuse functions are contracted with others of higher exponent in the former—unlike in the latter, where diffuse primitive functions are added to the basis set without contraction—is another important factor contributing to their improved performance, reflected in greater efficiency—that is, better convergence and more accurate results—in the energy minimization process.

The composition of (a)­σXZ0 for atoms from H to Ar is summarized in Table S1 of the Supporting Information (SI), which lists the number of exponents, primitive functions, and contracted functions. The number of contracted functions is identical between the (a)­σXZ0 and corresponding (a)­XZ basis sets, and the number of primitive functions is also very similar in both cases. Differences in the composition of the primitive functions in the reduced SIGMA BS, compared to the Dunning BS, depend on the size of the BS and are summarized in Table 1 where the differences in the number and type of primitive functions in (a)­σXZ0 with respect to (a)­XZ are collected. A file containing the reduced SIGMA basis sets for H to Ar, formatted for MOLPRO,^33–35^ is also included in the Supporting Information.

**Table 1:** Differencies in the Number and Type of Primitive Functions of (a)*σ*XZ0 with Respect to Dunning (a)­XZ Basis Sets

atoms	DZ	TZ	QZ
H	+ one *s*	+ one *s*	+ one *s*
He	+ one *s*	equal	equal
Li–Be	+ one *p*	– one *s* + one *p*	+ one *p*
B–Ne	+ one *p*	+ one *p*	+ one *p*
Na	+ one *p*	– one *s*	– three *s*
Mg	+ one *p*	equal	equal
Al–Ar	+ one *s*	+ one *p*	+ one *p*

### 2.2 Analysis of the Smallest Exponent per Shell

The σBS0 optimization process was carried out at the HF and CISD levels using MOLPRO.^35^ The states considered for F, Ne, Cl, and Ar are the same as those used in the optimization of the Dunning correlation-consistent BS. However, additional states were included for other elements, as shown in Table 2. For *a*σBS0, anionic and diatomic molecular states were also considered in the optimization.

**Table 2:** Reference States Used for the Optimization of SIGMA Basis Sets

States for optimization of σXZ0							
H ^2^S	He ^1^S						
H_2_ Σg+1							
Li ^2^S	Be ^1^S	B ^2^P	C ^3^P	N ^4^S	O ^3^P	F ^2^P	Ne ^1^S
Li ^2^P	Be ^3^P	B ^4^P	C ^1^D	N ^2^D	O ^1^D		
Li_2_ Σg+1			C ^1^S	N ^2^P	O ^1^S		
Na ^2^S	Mg ^1^S	Al ^2^P	Si ^3^P	P ^4^S	S ^3^P	Cl ^2^P	Ar ^1^S
Na ^2^P	Mg ^3^P	Al ^4^P	Si ^1^D	P ^2^D	S ^1^D		
Na_2_ Σg+1			Si ^1^S	P ^2^P	S ^1^S		

States added for optimization of aσXZ0							
H^– 1^S	He_2_ Σg+1						
Li^– 1^S	Be_2_ Σg+1	B^– 3^P	C^– 4^S	N^– 3^P	O^– 2^P	F^– 1^S	Ne_2_ Σg+1
Na^– 1^S	Mg_2_ Σg+1	Al^– 3^P	Si^– 4^S	P^– 3^P	S^– 2^P	Cl^– 1^S	Ar_2_ Σg+1

As will be demonstrated in the following section, (a)­σXZ0 yields lower energies than the corresponding (a)­XZ. A comparison of both BS shows that the smallest exponents for each shell in the standard σXZ0 and the corresponding XZ are highly similar. However, more significant differences arise in the augmented versions aσXZ0 and aXZ. A comparison of the smallest exponents in both sets is particularly noteworthy, and Figure 1 illustrates this comparison for the five angular momentum shells: *s*, *p*, *d*, *f*, and *g*.

**Figure 1:**
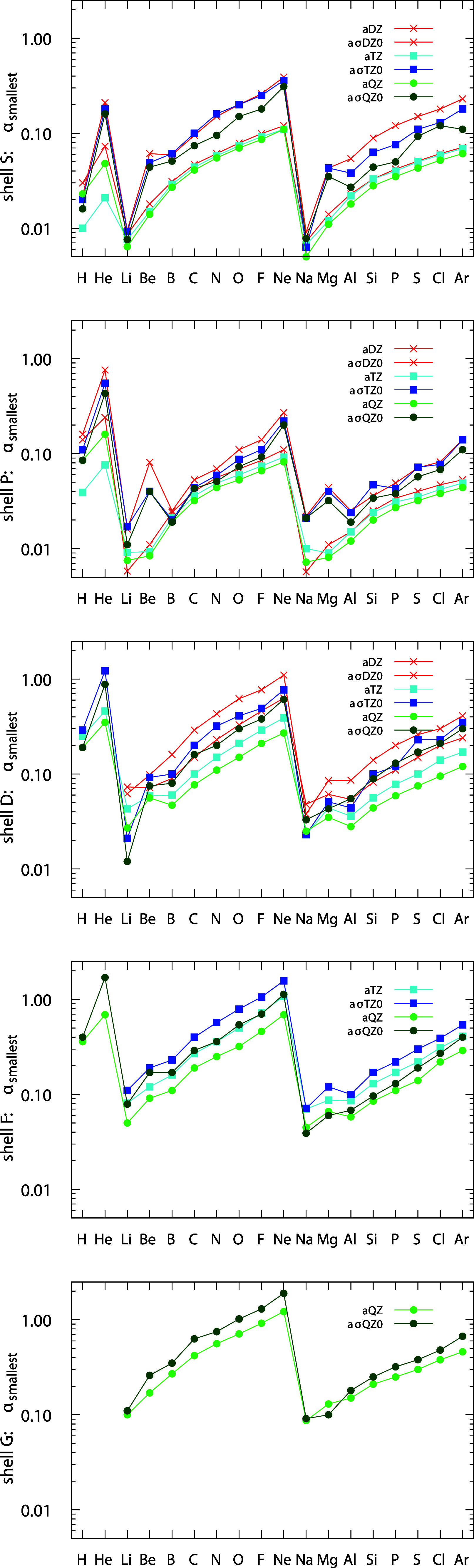
Smallest exponents for the aσXZ0 and aXZ basis sets from DZ to QZ sizes, and for the five angular momentum shells: *s*, *p*, *d*, *f*, and *g*.

Examining the five panels in Figure 1, the smallest exponents are higher for noble gases (He, Ne, and Ar) and lower for alkali metals (Li and K). It is clear that the exponents of the aσXZ0 are higher than those of the aXZ counterparts , and in both cases, they increase as the electron shells are filled and when moving from the *s* shell to the *g* shell. This trend is particularly evident in the aσQZ0 and aQZ sets (green), where the *s*-shell exponents are approximately ten times smaller than those of the *g* shell. In summary, while aXZ includes more diffuse primitive functions than the equivalent aσXZ0, lower exponents do not necessarily lead to a superior description of chemical systems, as will be shown in the following sections.

### 2.3 Atomic Energies

As outlined in Table 2, a comprehensive overview is provided regarding the states of atoms, anions, and dimers that were the focus of the (a)­σXZ0 optimization for elements ranging from H to Ar. Tables S2–S19 of the SI compile the raw atomic energies obtained for these atoms at the HF and CISD levels, along with the energy differences (in mEh) with respect to the equivalent aXZ, calculated as Δ*E* = *E*_aXZ_ – *E*_σXZ0_. Positive values of Δ*E* indicate that the (a)­σXZ0 yield lower energies and, thus, perform better from a variational perspective. Table S20 of the SI summarizes these energy differences using average values when multiple states are involved.

These differences are positive in all reported cases except in three HF energies (Li/Li_2_ with TZ and aTZ and Be with aTZ) and increase with atomic numbers with the lowest values observed for the alkali metals and the highest values for the noble gases. As expected, the differences decrease as the quality of the BS increases. These trends hold for both the standard and augmented versions of the BS, as well as for the HF and CISD energies.

The results are summarized graphically in Figure 2, which displays the average energy differences defined as Δ*E* = *E*_aXZ_ – *E*_σXZ0_. The left panels correspond to HF energies, and the right panels correspond to CISD correlation energies. In all cases—standard (Figure 2a and b) and augmented BS (Figure 2c and d)—the differences are positive and increase with atomic number across a period, with the largest difference observed for Ne and the smallest for Li. These findings confirm that, from a variational point of view, the (a)­σXZ0 outperforms the corresponding (a)­XZ.

**Figure 2:**
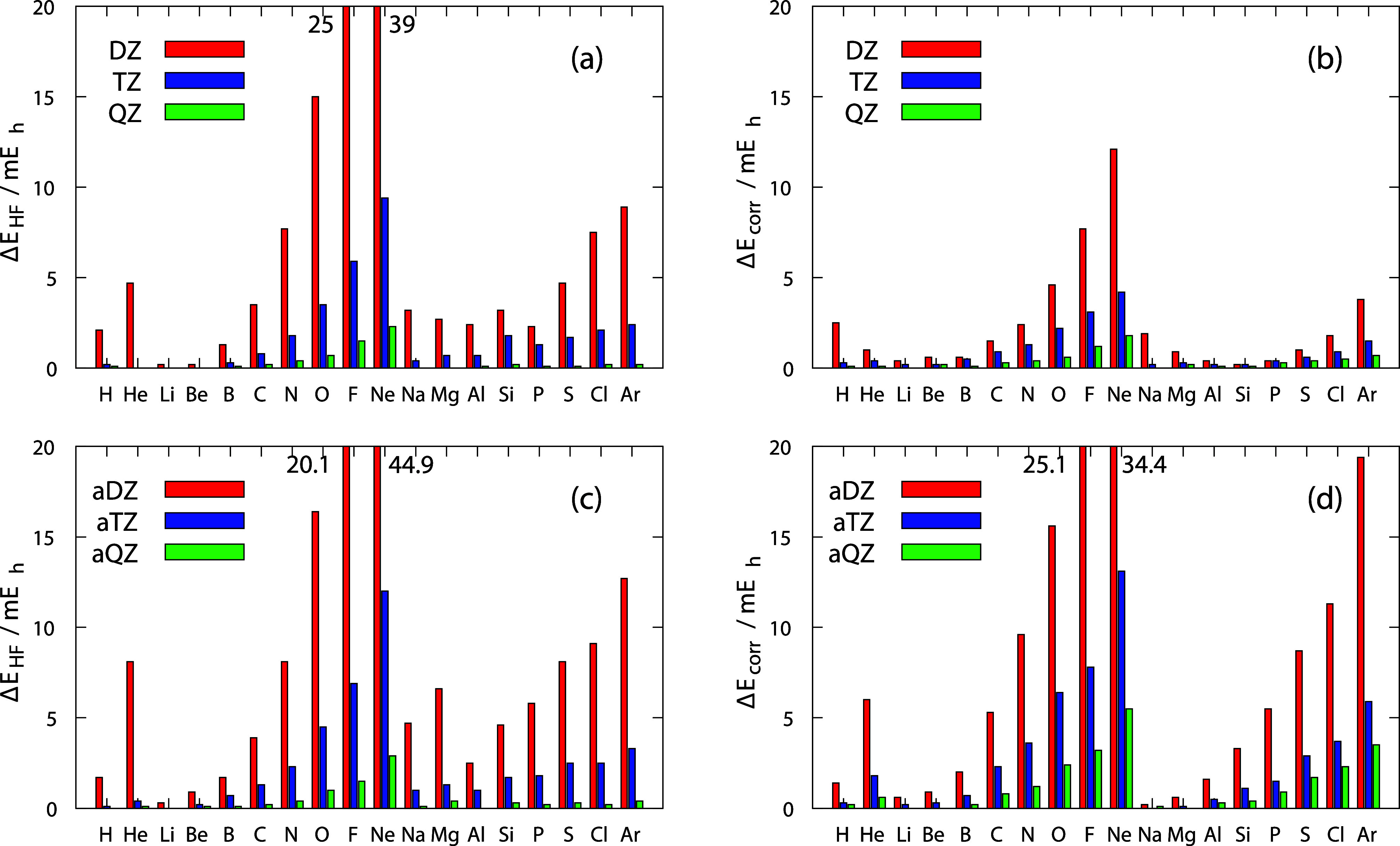
Energy differences between Dunning and SIGMA basis sets (Δ*E* = *E*_Dunning_ – *E*_SIGMA_) in mEh for atoms from H to Ar. (a) HF energy with standard basis set, (b) correlation energy and standard basis set, (c) HF energy with augmented basis set, (d) correlation energy with augmented basis set.

## 3 Computational Tests and Discussion

To test the performance of (a)­σXZ0, four types of studies have been carried out. In the first type, the results obtained with (a)­σXZ0 ranging from DZ to QZ were compared with those obtained using the corresponding (a)­XZ. In the remaining studies, only results obtained with the augmented BS are reported.

The first part of this section presents an extensive benchmark study of the reaction energies and geometries. The test set comprises 60 reactions involving small molecules containing first-, second-, and third-row atoms. A comparison of the two BS families across the set is made at the coupled cluster level with singles and doubles plus a quasi-perturbative correction for triple excitations [CCSD­(T)], using the larger Dunning a5Z set as a reference. In the remaining subsections, results obtained with different methods and augmented BS are reported to illustrate the good performance of *a*σBS0.

In the second test, we examine medium-sized systems of fixed geometry, using symmetry, at both the Density Functional Theory (DFT) level—specifically with B3LYP-D3—and the second-order Møller–Plesset perturbation theory (MP2) level. Calculations are performed on systems containing up to 62 atoms (pyrene, porphin, and tetra-benzene porphyrin). Similarly, we compute larger systems such as cucurbit[4]­uril and cucurbit[6]­uril macrocycles at the B3LYP and HF levels and perform timing comparisons.

The third test evaluates the feasibility of using resolution-of-the-identity (RI) techniques^36,37^ in place of conventional methods at the B3LYP and MP2 levels, applied to the reaction 2P_2_S_5_ → P_4_S_10_ as well as to calculations involving nitrogenous base pairs at the B3LYP-D3 and MP2 levels.

The final part of this section is dedicated to the calculation of three conformations of teixobactin, a peptide of biological interest with 186 atoms, at the RI-HF and RI-B3LYP-D3 levels.

### 3.1 Small Systems

The performance of σBS0 and *a*σBS0 on small systems has been analyzed using a data set of 60 reactions involving small atomic systems at the CCSD­(T) level of calculation, which is commonly considered the gold standard of ab initio quantum chemistry. Equilibrium distances and dissociation energies were computed, and the differences in the average equilibrium distances with respect to the a5Z, which is taken as a reference, are reported in Table S21 of the SI, along with the raw values of the reaction energies in kJ/mol. A statistical analysis of the differences with respect to the reference values is summarized in Table S22 of the SI. These processes were chosen because all species involved are closed-shell systems, thus avoiding artifacts associated with the selection of relevant states in open-shell cases. This ensures that differences in the results reflect only differences in BS performance.

The average differences with respect to the reference are plotted in Figure 3 for both geometries (left) and reaction energies (right panel). As can be seen, standard σXZ0 and XZ yield very similar relative errors for optimized geometries in these systems. However, in the case of augmented BS, the aXZ do not improve the geometries, while the aσXZ0 reduce the differences with respect to the reference by approximately 40% (see Figure 3a). For reaction energies, the standard σXZ0 offer little improvement over the corresponding XZ. Nevertheless, although the aXZ improve upon their standard counterparts by around 30%, the improvement achieved with the aσXZ0 reaches approximately 60% over the σXZ0 (see Figure 3b).

**Figure 3:**
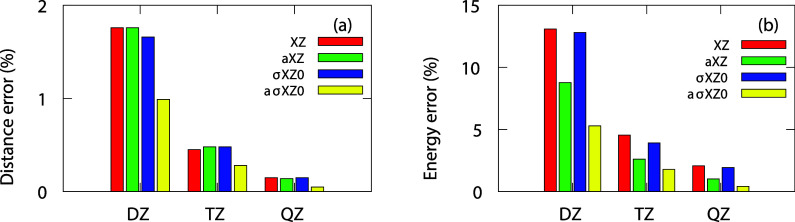
Statistical summary of 60 reactive systems computed at the CCSD­(T) level, with the 5Z or a5Z Dunning basis set as reference. (a) Distance errors; (b) reaction energy errors.

In summary, standard BS—both Dunning and reduced SIGMA—are insufficient to accurately describe these systems, and the use of augmented BS is essential. In this context, the performance of aσXZ0 clearly surpasses that of aXZ. Both total energies and geometries are significantly improved when moving from σXZ0 to aσXZ0, whereas no significant improvement in geometry is observed when transitioning from Dunning XZ to aXZ. For total energies, the improvement with aσTZ0 is such that the resulting energies are better than those obtained with the larger QZ.

### 3.2 Medium-Sized Systems

A second analysis of the (a)­σXZ0 performance was carried out on medium-sized molecular systems with up to 108 atoms, where calculations at the CCSD­(T) level are no longer feasible.

As a first example, we have analyzed the formation of tetra-benzene porphyrin (C_36_N_4_H_22_) from pyrene (C_16_H_10_) and porphin (C_20_N_4_H_12_), with reaction energies computed using different BS families as reported in Table 3. The molecular structures are displayed in Figure 4 and their geometries are reported in the SI. In particular, we compare the results obtained with Dunning’s (aug)-cc-pVXZ,^23,24^ Ahlrichs’ def2,^38,39^ Jensen’s aug-pcseg,^40–42^ aσXZ0, and Roos^43,44^ and Neese^45^ ANO BS. As can be seen, the convergence trends in reaction energies across the members of each family are quite similar for the SIGMA and ANO BS. In contrast, other families show discrepancies due to convergence issues. These can be resolved in the Dunning BS by adjusting the default program settings—specifically, by applying more stringent thresholds for integral accuracy and tighter convergence criteria—but prove fatal in the case of the larger members of Ahlrichs’ def2 and Jensen’s aug-pcseg BS. These convergence problems are likely due to a higher degree of quasi-linear dependence in those BS, particularly for the largest molecule, as shown in Table S23 of the SI, where the raw energies for reactants and products are listed. Furthermore, Table S24 of the SI reports the lowest overlap eigenvalue (LOE) and the number of eigenvalues below the 10^–7^ threshold (NEBT), showing that convergence issues occur in those BS with the highest number of small overlap eigenvalues. These are precisely the BS values in which diffuse primitive functions are added without contraction. On the other hand, no convergence issues arise with BS where diffuse functions are contracted with others, as is the case with the SIGMA and ANO BS.

**Figure 4:**
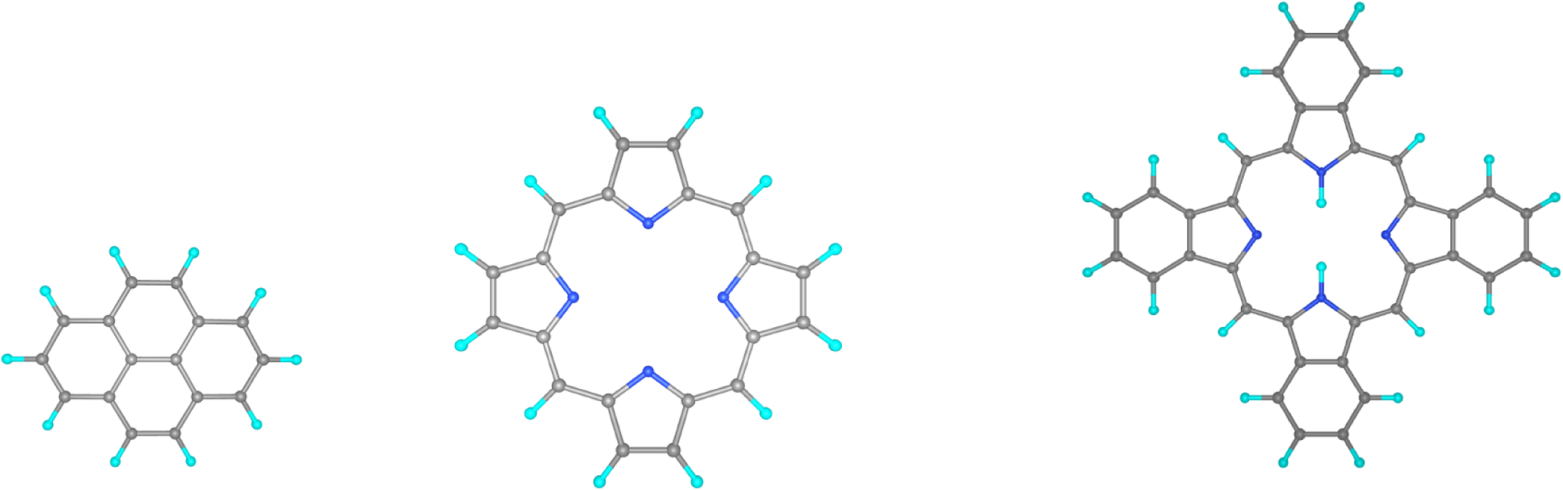
Geometric structures of pyrene, porphin, and tetra-benzene porphyrin.

**Table 3:** B3LYP-D3 and MP2 Reaction Energies (kj/mol) and Computational Time (hours) for the Reaction: C_16_H_10_ + C_20_N_4_H_12_ → C_36_N_4_H_22_

	ΔE: C_16_H_10_ + C_20_N_4_H_12_ → C_36_N_4_H_22_					
BS	#prim.	#cont.	B3LYP + D3	Time	MP2	Time
aDZ	1642	1118	552.4	4.1	683.2	4.8
aTZ	2870	2346	552.4	64	685.1	68
aQZ	4776	4212	522.2	206	696.3	376
def2-SVPD	1420	976	589.7	1.2	724.1	1.6
def2-TZVPPD	2498	1854	552.3	9.6	667.8	18
def2-QZVPPD	4112	3246	—^a^		—^a^	
apc1	1602	1118	555.2	4.4		
apc2	3052	2346	522.5	67		
apc3	5588	4478	—^a^			
aσDZ0	1784	1118	544.8	1.6	668.1	2.2
aσTZ0	3012	2346	553.5	15	676.2	19
aσQZ0	4918	4212	552.2	130	673.2	165
anoaDZR	2880	1118	546.2	8.1	688.4	10
anoaTZR	4050	2346	555.1	51	680.8	56
anoaDZN	3412	1118	539.6	5.7	648.2	7.4
anoaTZN	4972	2386	552.4	30	668.3	37
anoaQZN	6514	3900	551.8	185	669.9	222

a Not converged.

In addition, Table 3 reports the computational time measured on a system equipped with an Intel Xeon Platinum 8362 CPU @ 2.80 GHz. As expected, the computational cost is lowest for Ahlrichs’ BS, which contain the fewest contractions. However, although def2-TZVPPD may appear to be the most satisfactory choice, the poorer performance of def2-SVPD and the lack of convergence of def2-QZVPPD suggest that this may not be the case for other systems. In all other cases—where the number of contractions is comparable and the quality of the results is similar—the aσXZ0 basis sets yield shorter computational times than their counterparts from other basis set families.

Performance on larger systems was evaluated for cucurbit[4]­uril (CB[4]) and cucurbit[6]­uril (CB[6]) macrocycles,^46^ which exhibit D_2h_ symmetry and contain up to 108 atoms. Geometries are displayed in Figure 5 and the coordinates are reported in the SI. Cucurbit­[*n*]­urils (CB­[*n*]) are a family of macrocyclic compounds obtained from the condensation of formaldehyde and glycoluril.^47^ CB­[*n*] compounds are cage-like host molecules with a rigid hydrophobic cavity and have attracted significant interest due to their ability to form inclusion complexes with a wide range of molecules, including drugs, organic compounds, and biomolecules.^48^ They have been extensively studied in areas such as catalysis, drug delivery, electrochemistry, photochemistry, and more.^49,50^

**Figure 5:**
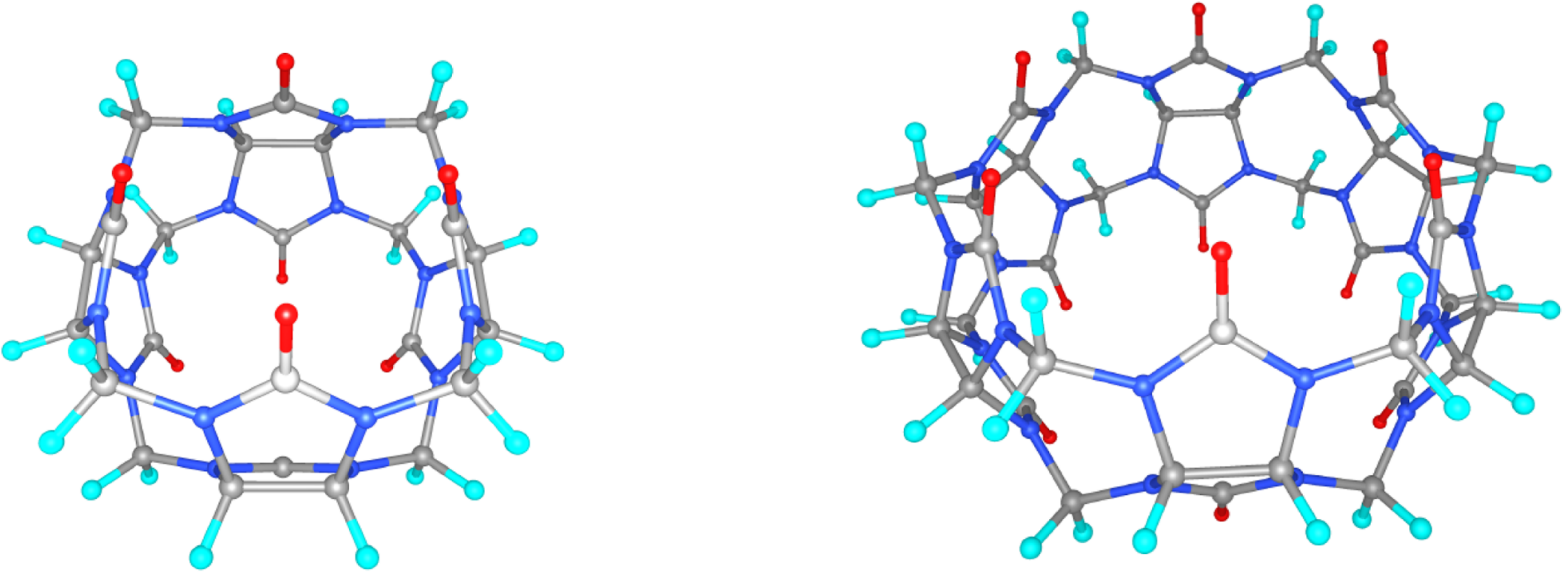
Geometric structures of macrocycles CB[4] and CB[6].

These systems were computed at the B3LYP and HF levels using the aXZ and aσXZ0, and as in previous cases, geometries were kept fixed. Computational times were measured with integrals computed on the fly in all cases. As shown in Table 4, the energies obtained with aσXZ are lower than those with aXZ, while the computational cost is also reduced. Using the default settings of the MOLPRO program, the calculations for the larger CB[6] system with aQZ did not satisfy the convergence criteria within a sufficiently large number of cycles. By contrast, convergence with the aσQZ0 under the same default settings was perfectly satisfactory. Once again, the convergence issues appear to be related to an insufficient degree of linear independence in the BS, as illustrated in Table S25 of the SI, where the lowest overlap eigenvalues (LOEs) are reported. The LOEs for the aXZ are shown to be 2 orders of magnitude smaller than those of their aσXZ0 counterparts.

**Table 4:** B3LYP and HF Calculations of Cucurbit[4] and Cucurbit[6] Urils: Total Energies in Eh and Computational Time in hours

	CB[4] C_24_N_16_O_8_H_24_					
BS	#prim.	#cont.	B3LYP	Time	HF	Time
aDZ	1984	1320	–2405.956300	2.8	–2393.363489	3.0
aTZ	3384	2760	–2406.502781	28.5	–2393.885047	25.8
aQZ	5616	4944	–2406.672812	258	–2394.027167	230
aσDZ0	2112	1320	–2406.393567	1.9	–2393.769647	1.9
aσTZ0	3552	2760	–2406.641028	15	–2393.997062	13
aσQZ0	5784	4944	–2406.710610	141	–2394.048585	140

	CB[6] C_36_N_24_O_12_H_36_					
BS	#prim.	#cont.	B3LYP	Time	HF	Time
aDZ	2916	1980	–3609.008535	9.4	–3590.123438	10.0
aTZ	5076	4140	–3609.829254	85.1	–3590.906257	84.9
aQZ	8424	7416	not converged	>800	not converged	>800
aσDZ0	3168	1980	–3609.666507	4.5	–3590.733542	5.0
aσTZ0	5328	4140	–3610.036536	43.5	–3591.074121	37.1
aσQZ0	8676	7416	–3610.141033	382	–3591.151761	339

The superior performance of aσXZ over aXZ is illustrated in Figure 6, which displays the ratio of computational times between aQZ and aσQZ for the five systems analyzed in this section. The reduction in computational cost is particularly notable for the largest compound, where the time is reduced by more than a factor of 2.

**Figure 6:**
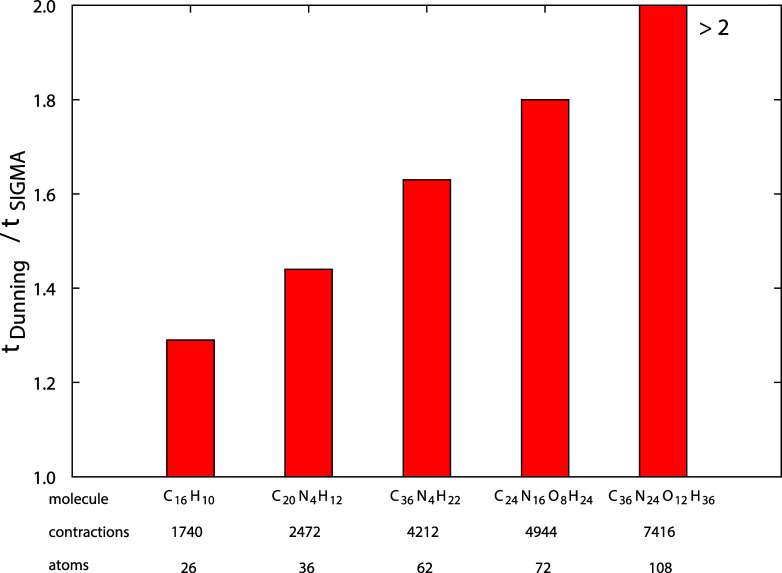
Comparison of Dunning/SIGMA QZ basis set computational times at the DFT level for pyrene, porphin, tetra-benzene porphyrin, CB[4], and CB[6].

### 3.3 RI Calculations

The performance of aσXZ0 has also been tested using the RI approximation at the B3LYP and MP2 levels for the reaction 2 P_2_S_5_ → P_4_S_10_. As is well known, this effective and powerful approach reduces the computational cost of large-scale electronic structure calculations by using auxiliary functions.^51^ In the present work, we used the same auxiliary basis sets employed for aXZ in the RI-DFT and RI-MP2 calculations with aσXZ0. In particular, the JKFit and MP2Fit sets,^52^ commonly used with aXZ, were utilized as implemented in MOLPRO, demonstrating that, in principle, the aσXZ0 basis sets do not require newly developed auxiliary sets specifically tailored for them.

Table 5 reports the RI energies in Eh for P_2_S_5_ and P_4_S_10_, along with the reaction energy in kJ/mol, the average relative errors of interatomic distances (for distances shorter than 3 Å) with respect to the a5Z reference, and the computational time. As can be seen, the reaction energies and computational costs are very similar for both types of BS, while the absolute energy values and average geometric deviations are lower with *a*σBS0. The results obtained without the RI approximation are provided in Table S26 of the SI.

**Table 5:** RI-B3LYP and RI-MP2 Calculations for the Reaction 2 P_2_S_5_ → P_4_S_10_, Total Energies in Eh, Reaction Energies in kj/mol, Average Relative Error in Distance for Distances Smaller Than 3 Å, and Computational Time in seconds

BS	#prim.	#cont.	RI-B3LYP-P_2_S_5_	RI-B3LYP-P_4_S_10_	Δ*E*_RI_	%dis. Av. err.	Time RI	
aDZ	700	378	–2673.449566	–5346.962470	166.29	1.671 1.737	16	126
aTZ	1050	700	–2673.555703	–5347.173409	162.79	0.619 0.679	47	373
aQZ	1568	1176	–2673.587413	–5347.237654	164.96	0.254 0.277	126	1066
a5Z	2268	1834	–2673.619252	–5347.303034	169.42	reference	330	3109
aσDZ0	742	378	–2673.530442	–5347.118189	150.45	0.762 0.778	16	139
aσTZ0	1092	700	–2673.581118	–5347.224180	162.63	0.339 0.359	46	361
aσQZ0	1610	1176	–2673.609938	–5347.286540	175.06	0.066 0.090	131	1074

BS	#prim.	#cont.	RI-MP2-P_2_S_5_	RI-MP2-P_4_S_10_	Δ*E*_RI_	%dis. Av. err.	Time RI	
aDZ	700	378	–2670.144763	–5340.400425	291.17	2.294 2.380	14	119
aTZ	1050	700	–2670.488827	–5341.101829	326.02	0.890 0.901	37	366
aQZ	1568	1176	–2670.607747	–5341.342290	332.90	0.324 0.325	103	1080
a5Z	2268	1834	–2670.661410	–5341.452930	341.60	reference	295	3162
aσDZ0	742	378	–2670.268922	–5340.636485	258.98	1.275 1.153	14	105
aσTZ0	1092	700	–2670.532366	–5341.186196	318.90	0.472 0.388	43	361
aσQZ0	1610	1176	–2670.630293	–5341.388363	335.48	0.035 0.067	123	1156

The small differences observed between the results with and without the RI approach are noteworthy. The largest relative deviations (ranging from 0.4% to 1.1%) in the reaction energies appear in calculations performed with double-ζ BS.

In addition, RI-B3LYP-D3 and RI-MP2 calculations have been carried out using the augmented BS for weak interactions between pyrimidine bases—adenine + thymine, guanine + cytosine, and adenine + uracil—that occur in the double DNA helix and in the single strand of RNA.^53^ The optimized geometries of these base pairs are depicted in Figure 7.

**Figure 7:**
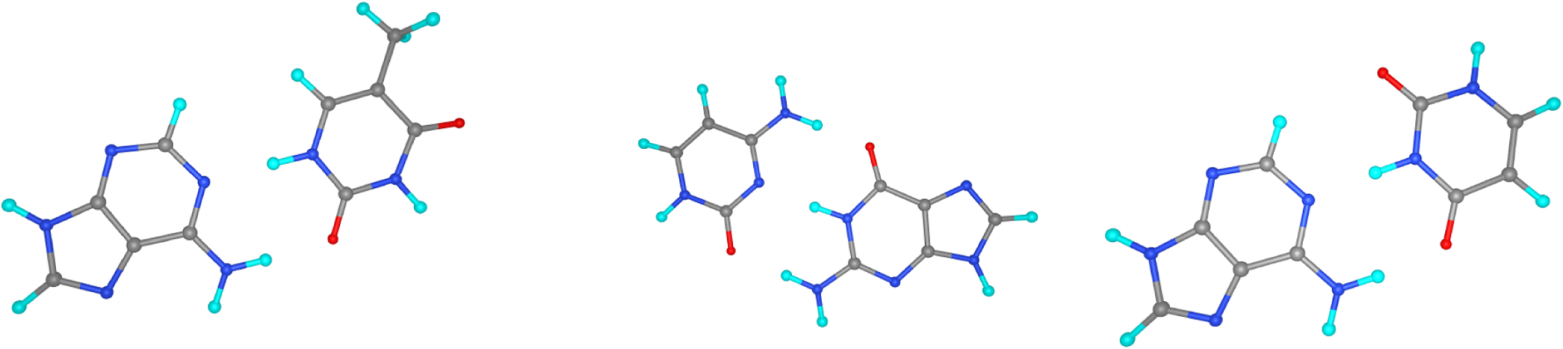
Geometric structures of nitrogenous base pairs: adenine-thymine, guanine-cytosine, and adenine-uracil.

The results are presented in Table 6, with an emphasis on the performance of the RI calculations. Once again, there is clear agreement between the reaction energies computed with (Δ_RI_) and without (Δ) the RI approximation for both types of BS. Moreover, the geometries optimized with aσXZ0 are closer to the reference values (a5Z for B3LYP-D3 and aQZ for MP2) than those optimized with the corresponding aXZ sets. Table S27 of the SI reports the raw energy values, the differences between the energies computed with and without RI, and the deviations in interatomic distances shorter than 3 Å with respect to the a5Z reference. As can be seen, the energies obtained with aσXZ0 are closer to the reference than those obtained with aXZ, and the average geometric deviations relative to the reference are also smaller. Finally, the differences between RI and conventional total energies are very small and have a negligible impact on the reaction energies.

**Table 6:** RI-B3LYP-D3 and RI-MP2 Calculations for Nitrogenous Base Pairs: Total Energies in Eh, Energy Errors (RI Minus Conventional Method Energies) in kj/mol, RI Computation Times in seconds, Interaction Energies from Both RI and Conventional Methods in kj/mol, and Average Relative Distance Errors for Interatomic Distances Below 3 Å

	Adenine + Thymine							
BS	#prim.	#cont.	RI-B3LYP+D3	err RI	Time RI	Δ*E*_RI_	Δ*E*	%dis. Av. err.
aDZ	786	536	–921.139345	0.40	195	76.10	76.11	0.534
aTZ	1377	1127	–921.356107	0.07	650	71.78	71.78	0.056
aQZ	2295	2026	–921.420248	0.08	2493	70.92	70.88	0.014
a5Z	3649	3293	–921.438597	0.06	7499	70.81	70.76	reference
aσDZ0	854	536	–921.308524	0.25	182	72.75	72.75	0.209
aσTZ0	1445	1127	–921.407062	0.15	645	71.83	71.83	0.035
aσQZ0	2363	2026	–921.434617	0.05	2526	71.16	71.17	0.008

BS	#prim.	#cont.	RI-MP2	err RI	Time RI	Δ*E*_RI_	Δ*E*	%dis. Av. err.
aDZ	786	536	–919.097455	5.35	195	78.36	78.27	0.896
aTZ	1377	1127	–919.870121	2.58	823	74.83	74.78	0.146
aQZ	2295	2026	–920.123910	0.95	4018	71.63	71.62	reference
aσDZ0	854	536	–919.438121	6.55	196	76.45	76.36	0.384
aσTZ0	1445	1127	–919.982024	2.64	832	72.96	72.92	0.059
aσQZ0	2363	2026	–920.158182	0.92	4162	71.29	71.22	reference

	Guanine + Cytosine							
BS	#prim.	#cont.	RI-B3LYP+D3	err RI	Time RI	Δ*E*_RI_	Δ*E*	%dis. Av. err.
aDZ	775	527	–937.196941	0.47	189	130.51	130.54	0.526
aTZ	1352	1104	–937.415824	0.04	639	124.86	124.86	0.060
aQZ	2247	1980	–937.481156	0.07	2439	123.72	123.72	0.014
a5Z	3556	3213	–937.499688	0.06	6430	123.37	123.35	reference
aσDZ0	842	527	–937.367429	0.33	182	126.35	126.35	0.216
aσTZ0	1419	1104	–937.467617	0.11	634	125.32	125.32	0.039
aσQZ0	2314	1980	–937.495730	0.05	2401	124.22	124.22	0.009
BS	#prim.	#cont.	RI-MP2	err RI	Time RI	Δ*E*_RI_	Δ*E*	%dis. Av. err.
aDZ	775	527	–935.134281	5.43	159	126.74	126.66	0.896
aTZ	1352	1104	–935.918643	2.62	792	124.37	124.30	0.147
aQZ	2247	1980	–936.177168	0.88	3928	121.09	121.00	reference
aσDZ0	842	527	–935.480041	6.58	163	124.48	124.39	0.348
aσTZ0	1419	1104	–936.032301	2.65	799	122.50	122.46	0.059
aσQZ0	2314	1980	–936.211933	0.94	4012	120.58	120.32	reference

	Adenine + Uracile							
BS	#prim.	#cont.	RI-B3LYP+D3	err RI	Time RI	Δ*E*_RI_	Δ*E*	%dis. Av. err.
aDZ	729	495	–881.841931	0.42	144	72.85	72.85	0.557
aTZ	1269	1035	–882.047775	0.05	511	68.24	68.24	0.056
aQZ	2106	1854	–882.109127	0.06	1954	67.35	67.35	0.016
a5Z	3339	3006	–882.126514	0.04	5420	66.93	66.91	reference
aσDZ0	792	445	–882.002552	0.29	144	69.70	69.69	0.232
aσTZ0	1332	1035	–882.096482	0.12	511	68.51	68.51	0.040
aσQZ0	2169	1854	–882.122853	0.05	1943	67.83	67.84	0.009

BS	#prim.	#cont.	RI-MP2	err RI	Time RI	Δ*E*_RI_	Δ*E*	%dis. Av. err.
aDZ	729	495	–879.901395	5.13	153	74.59	74.51	0.885
aTZ	1269	1035	–880.636825	2.49	667	71.02	70.97	0.149
aQZ	2106	1854	–880.879485	0.78	2956	67.86	67.84	reference
aσDZ0	792	445	–880.2268726	6.29	155	72.27	72.18	0.393
aσTZ0	1332	1035	–880.7439290	2.52	642	69.11	69.08	0.065
aσQZ0	2169	1854	–880.9123523	1.06	2678	67.50	67.48	reference

### 3.4 Large Systems

The performance on large systems has been tested using the teixobactin peptide, which consists of 186 atoms and is of biological interest as an antibiotic agent.^54^ These large biological systems involve numerous intramolecular interactions, particularly hydrogen bonds, making the use of augmented BS highly advisable for accurate descriptions. The teixobactin peptide conformations were generated^55^ using a Monte Carlo approach and optimized with the MMFF force field. Of the 20 conformations available, three were selected for this analysis: the most compact (#1), the most extended (#20), and an intermediate one (#10). The geometries of these conformations are listed in Figure 8. The reference relative energies of the most compact conformers, #1 and #10, with respect to the most extended one, #20, are, respectively, −222.8 and +39.1 kJ/mol at the MW7/B3LYP-D3 level.^55^

**Figure 8:**
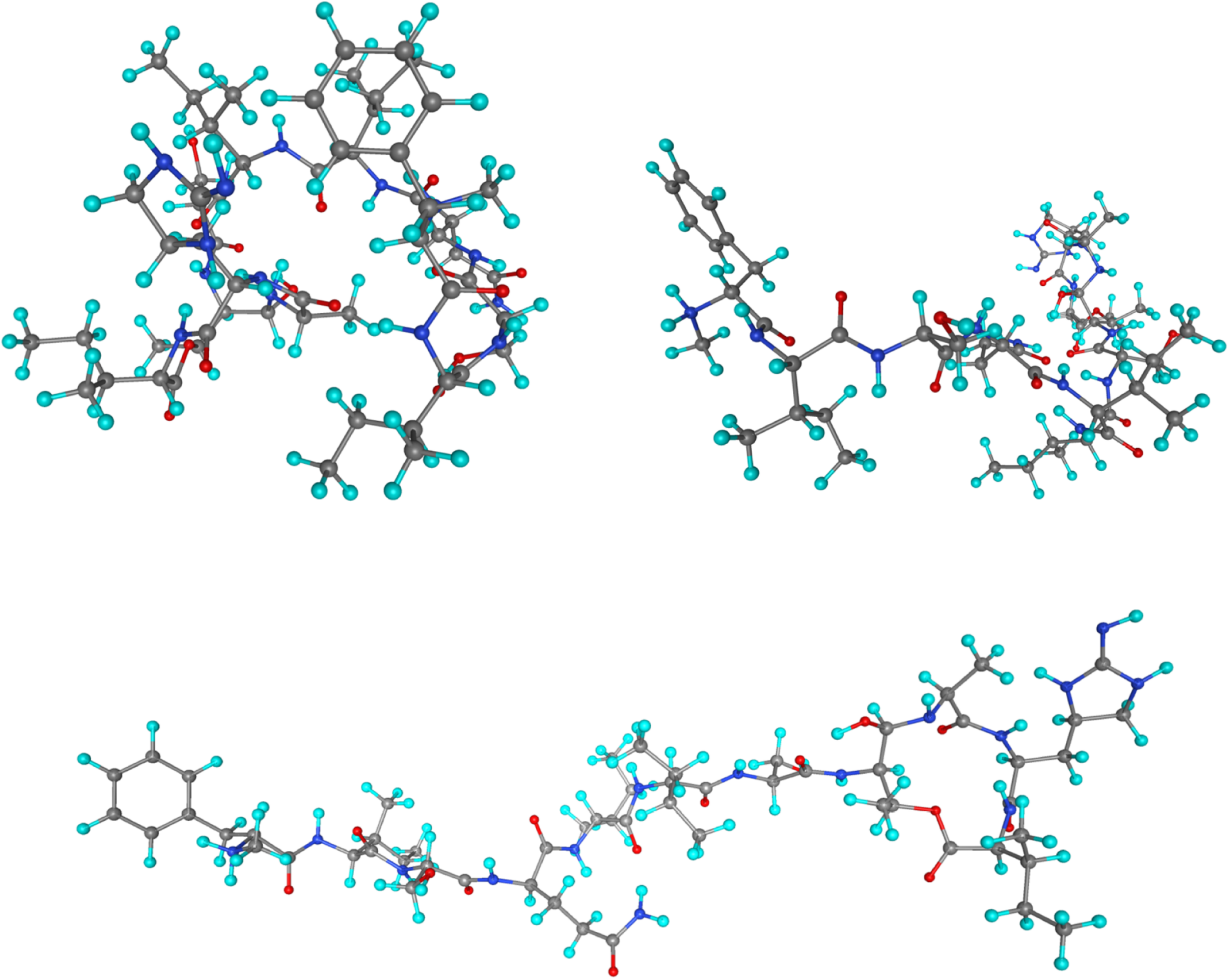
Optimized geometries of conformations 1, 10, and 20 of the teixobactin peptide. Coordinates taken from ref. 44.

RI-HF and RI-B3LYP-D3 calculations for the three conformations using aDZ and *a*σDZ0 BS are reported in Table 7. It can be observed that total energies are lower and closer to the reference when using aσDZ0 compared to aDZ. Furthermore, although the aσXZ0 contain approximately 10% more primitive functions than the aDZ, the computational times are similar or even lower with aσXZ0, depending on the conformer’s shape. Specifically, the computational time with aXZ is higher for compact systems than for extended ones, while it remains relatively independent of system shape when *a*σBS0 are employed.

**Table 7:** RI-HF and RI-B3LYP-D3 Calculations of Three Conformations of Teixobactin: Total Energies in Eh, Stabilization Energy in kj/mol and Computational Time in hours

aDZ	RI-HF	ΔE	Time	RI-B3LYP-D3	ΔE	Time
Conf 1	–4193.650313	–60.9	22.3	–4217.227108	–270.2	33.6
Conf 10	–4193.590937	+95.0	19.2	–4217.114393	+25.7	22.4
Conf 20	–4193.627101	0.0	17.6	–4217.124195	0.0	19.8

aσDZ0	RI-HF	ΔE	Time	RI-B3LYP-D3	Δ*E*	Time
Conf 1	–4194.405342	–50.5	18.9	–4218.052688	–237.7	19.7
Conf 10	–4194.347556	+101.2	18.3	–4217.949651	+32.9	20.4
Conf 20	–4194.386103	0.0	17.5	–4217.962169	0.0	18.3

Reference^a^	HF	ΔE		B3LYP-D3	ΔE	
Conf 1	–4194.881387	–24.9		–4220.804174	–222.8	
Conf 10	–4194.829751	+110.7		–4220.748471	+39.1	
Conf 20	–4194.871919	0.0		–4220.782565	0.0	

a Extracted from ref. 44.

## 4 Conclusions

A new family of SIGMA BS, named reduced SIGMA BS, has been reported with sizes ranging from DZ to QZ, in both standard and augmented versions, and covering atoms from H to Ar. The main difference with respect to previous SIGMA BS lies in the reduction of the number of primitive functions and the elimination of the restriction that exponents of primitive functions appearing in a shell with a given *L* must also be present in all shells with *l* <  *L*.

The construction of the reduced SIGMA BS follows the matryoshka doll scheme previously reported. In the augmented version, diffuse functions with exponents higher than those in the corresponding Dunning BS are added. In the *s* and *p* shells, these diffuse functions are incorporated into the contractions, whereas in the remaining shells, they are added without contraction. Furthermore, unlike in the Dunning augmented BS, all exponents of the primitive functions in aσXZ0 are reoptimized.

In atomic calculations, the (a)­σXZ0 BS outperform the (a)­XZ of similar size from a variational point of view, with the difference being more pronounced in the augmented BS. In molecular calculations, the performance of the standard σXZ0 and XZ sets is very similar; however, the *a*σBS0 outperform the corresponding aXZ at the HF, CCSD­(T), and DFT levels, both with direct computation of the integrals and when using RI techniques. In some cases, the energy obtained with aσXZ is lower than that attained with the (X + 1)­Z set, despite the larger size of the latter. Furthermore, the aσXZ0 sets also perform better than aXZ in geometry optimizations, which can be attributed to the better description of the valence shell with contractions containing diffuse primitive functions compared to the noncontracted primitive functions directly added in the Dunning BS.

In medium-sized and large compact systems, the computational cost with *a*σBS0 at the RI-HF and RI-B3LYP-D3 levels is lower than that with the equivalent aXZ in the tested systems, with a time ratio ranging from 1.2 in the smallest molecule (pyrene) to more than two in the largest (CB[6]). Additionally, energy optimization convergence with *a*σBS0 is satisfactory in all cases, regardless of the system size. In contrast, convergence issues have been observed at the MP2 level with aTZ and aQZ in large, compact systems such as tetra-benzene porphyrin. These issues are likely due to quasi-linear dependencies in the BS, as suggested by the presence of very small eigenvalues in the overlap matrix—approximately 2 orders of magnitude smaller with aTZ and aQZ than with aσTZ0 and aσQZ0. In this context, molecular planarity also appears to contribute to the convergence difficulties encountered with aTZ and aQZ in the systems under study.

In summary, the standard versions of XZ and σXZ0 perform very similarly in molecular calculations, but the augmented version aσXZ0 clearly outperforms its corresponding aXZ counterpart—both in energy and geometry optimization as well as in computational cost and convergence behavior—especially when large, compact systems are involved.

## Supplementary Material







## Notes

File *Supplementary_Tables.pdf* contains 27 tables (PDF)
File *geometries.txt* contains geometries of compounds in Figures 4 and 5 (TXT)
File *sigma-BS.txt* contains the aσXZ0 BS in Molpro format (TXT)
