# Supplementary material for: Reduced Serially Improved GTOs for Molecular Applications from H to Ar: Efficient Diffuse Functions in Augmented Basis Sets
Source: J Phys Chem A. 2025 Nov 24;129(48):11232–44. doi: 10.1021/acs.jpca.5c05079 (PMC12683629; doi:10.1021/acs.jpca.5c05079)
Supplement: Supplementary file 3 [file jp5c05079_si_003.pdf]

Supplementary Tables to: Reduced Serially Improved GTOs for Molecular Applications from H to Ar. Efficient Diffuse Functions in Augmented Basis Sets

Ignacio Ema, Jesús San-Fabián, Guillermo Ramírez,
Rafael López, and José Manuel García de la Vega
*Departamento de Química Física Aplicada. Universidad Autónoma de Madrid. E-28049. Madrid. Spain**

* nacho.ema@uam.es

TABLES CONTENTS

TABLE S1: Composition SIGMA basis sets for atoms H to Ar.

TABLES S2 to S19: HF and CISD energies computed in the optimization of the basis sets from H to Ar.

TABLE S20: HF and CISD average energy diferences (a)XZ and (a)σXZ0 basis sets.

TABLE S21: Differences in the average relative equilibrium distances and reaction energies for reactants and products of several closed-shell reactions.

TABLE S22: Statistical nalysis of differences in the average equilibrium distances and reaction energies.

TABLE S23: MP2 and B3LYP-D3 energies for the compounds of the reaction: $C_{16}H_{10}$ + $C_{20}N_4H_{12} \rightarrow C_{36}N_4H_{22}$

TABLE S24: Lowest Overlap Eigenvalues and Number of Eigenvalues Below 10^{-7} threshold for the reactants and product of the reaction: $C_{16}H_{10} + C_{20}N_4H_{12} \rightarrow C_{36}N_4H_{22}$

TABLE S25: Lowest Overlap Eigenvalues for cucurbit[4] and cucurbit[6] urils.

TABLE S26: B3LYP and MP2 calculations for ground states of reactant and product of reaction: 2 $P_2S_5 \rightarrow P_4S_{10}$. RI vs conventional calculations.

TABLE S27: RI-B3LYP-D3 and RI-MP2 calculations for Adenine, Thymine, Guanine, Cytosine and Uracil.

TABLE S1: Composition SIGMA basis sets for atoms H to Ar. Number of primitive and contracted functions.

BS	# Primitives	# Contractions
	First row: H and He.	
σ0DZ	8 (5s, 1p)	5 [2s, 1p]
aσ0DZ	12 (6s, 2p)	9 [3s, 2p]
σ0TZ	17 (6s, 2p, 1d)	14 [3s, 2p, 1d]
aσ0TZ	26 (7s, 3p, 2d)	23 [4s, 3p, 2d]
σ0QZ	33 (7s, 3p, 2d, 1f)	30 [4s, 3p, 2d, 1f]
aσ0QZ	49 (8s, 4p, 3d, 2f)	46 [5s, 4p, 3d, 2f]
	Second row: Li to Ne.	
σ0DZ	29 (9s, 5p, 1d)	14 [3s, 2p, 1d]
aσ0DZ	38 (10s, 6p, 2d)	23 [4s, 3p, 2d]
σ0TZ	45 (10s, 6p, 2d, 1f)	30 [4s, 3p, 2d, 1f]
aσ0TZ	61 (11s, 7p, 3d, 2f)	46 [5s, 4p, 3d, 2f]
σ0QZ	71 (12s, 7p, 3d, 2f, 1g)	55 [5s, 4p, 3d, 2f, 1g]
aσ0QZ	96 (13s, 8p, 4d, 3f, 2g)	80 [6s, 5p, 4d, 3f, 2g]
	Third row: Na to Ar.	
σ0DZ	44 (12s, 9p, 1d)	18 [4s, 3p, 1d]
aσ0DZ	53 (13s, 10p, 2d)	27 [5s, 4p, 2d]
σ0TZ	62 (15s, 10p, 2d, 1f)	34 [5s, 4p, 2d, 1f]
aσ0TZ	78 (16s, 11p, 3d, 2f)	50 [6s, 5p, 3d, 2f]
σ0QZ	90 (16s, 12p, 3d, 2f, 1g)	69 [6s, 5p, 3d, 2f, 1g]
aσ0QZ	115 (17s, 13p, 4d, 3f, 2g)	84 [7s, 6p, 4d, 3f, 2g]

TABLE S2: H, H^- and H_2 energies (E_h) at the HF and CISD levels used in the basis set optimization for Hydrogen atom and ΔE (mE_h) = E(XZ) - E(σXZ) in parentheses.

	$H(^2S)$	$H^-(^1S)$		$H_2(^1\Sigma_g^+$ 1.4 au)	
	HF	HF	CISD	HF	CISD
σDZ0	-0.499723 (0.445)			-1.150754 (3.846)	-1.168041 (4.643)
σTZ0	-0.499840 (0.030)			-1.151877 (0.458)	-1.172861 (0.526)
σQZ0	-0.499975 (0.030)			-1.152093 (0.115)	-1.173943 (0.147)
aσDZ0	-0.499822 (0.488)	-0.487222 (0.441)	-0.525236 (1.208)	-1.151111 (4.127)	-1.169634 (5.026)
aσTZ0	-0.499942 (0.121)	-0.487400 (-0.240)	-0.526845 (0.282)	-1.151945 (0.472)	-1.173228 (0.595)
aσQZ0	-0.499971 (0.023)	-0.487623 (-0.185)	-0.527420 (0.281)	-1.152087 (0.098)	-1.173999 (0.132)

TABLE S3: He and He_2 energies (E_h) at the HF and CISD levels used in the basis set optimization for Helium atom and ΔE (mE_h) = E(XZ) - E(σXZ) in parentheses.

	He (^{1}S)		He_2($^1\Sigma_g^+$ 5.94 au)	
	HF	CISD	HF	CISD
σDZ0	-2.859892 (4.732)	-2.893225 (5.660)		
σTZ0	-2.861150 (-0.003)	-2.900626 (0.400)		
σQZ0	-2.861509 (-0.005)	-2.902540 (0.139)		
aσDZ0	-2.861148 (5.444)	-2.899005 (9.456)	-5.707685 (10.796)	-5.783390 (18.821)
aσTZ0	-2.861511 (0.328)	-2.902178 (1.580)	-5.708475 (0.570)	-5.790286 (2.865)
aσQZ0	-2.861622 (0.100)	-2.903048 (0.515)	-5.708704 (0.118)	-5.792262 (0.833)

TABLE S4: Li, Li^- and Li_2 energies (E_h) at the HF and CISD levels used in the basis set optimization for Lithium atom and ΔE (mE_h) = E(XZ) - E(σXZ) in parentheses.

	Li(^{2}S, ^{2}P)	Li^-(^{1}S)		Li_2($^1\Sigma_g^+$ 5.1 au)	
	HF	HF	CISD	HF	CISD
σDZ0	-7.432401 (-0.019)			-14.870085 (0.410)	-14.901218 (0.565)
	-7.364715 (0.130)				
σTZ0	-7.432613 (-0.065)			-14.871464 (-0.027)	-14.903397 (0.193)
	-7.364884 (-0.104)				
σQZ0	-7.432694 (-0.001)			-14.871643 (0.011)	-14.903742 (0.022)
	-7.365030 (0.003)				
aσDZ0	-7.432415 (-0.010)	-7.427811 (0.423)	-7.455032 (0.834)	-14.870440(0.406)	-14.901930 (0.448)
	-7.364762 (0.155)				
aσTZ0	-7.432624 (-0.058)	-7.428018 (-0.127)	-7.455260 (0.239)	-14.871508(-0.038)	-14.903499 (0.217)
	-7.364969 (-0.023)				
aσQZ0	-7.432696 (0.000)	-7.428136 (-0.048)	-7.455380 (0.115)	-14.871646(0.011)	-14.903764 (0.026)
	-7.365037 (0.009)				

TABLE S5: Be and Be_2 energies (E_h) at the HF and CISD levels used in the basis set optimization for Beryllium atom and ΔE (mE_h) = E(XZ) - E(σXZ) in parentheses.

	Be (^{1}S, ^{3}P)		Be_2($^1\Sigma_g^+$ 4.8 au)	
	HF	CISD	HF	CISD
σDZ0	-14.572302 (-0.035)	-14.617711 (0.939)		
	-14.510658 (0.441)	-14.516427 (0.739)		
σTZ0	-14.572539 (-0.335)	-14.618377 (0.620)		
	-14.511012 (-0.189)	-14.517946 (0.645)		
σQZ0	-14.572951 (-0.017)	-14.618930 (0.023)		
	-14.511404 (-0.004)	-14.518552 (0.461)		
aσDZ0	-14.572604 (0.223)	-14.618353 (1.450)	-29.231117 (1.841)	-29.237471 (2.795)
	-14.511085 (0.769)	-14.517892 (1.260)		
aσTZ0	-14.572867 (-0.009)	-14.618845 (0.408)	-29.231672 (0.446)	-29.238925 (0.692)
	-14.511343 (0.125)	-14.518473 (0.316)		
aσQZ0	-14.572995 (0.026)	-14.619057 (0.098)	-29.231933 (0.100)	-29.239456 (0.195)
	-14.511472 (0.061)	-14.518661 (0.104		

TABLE S6: B and B^- energies (E_h) at the HF and CISD levels used in the basis set optimization for Boron atom and ΔE (mE_h) = E(XZ) - E(σXZ) in parentheses.

	B (^2P, ^4P)		B^- (^3P)	
	HF	CISD	HF	CISD
σDZ0	-24.527412 (0.848)	-24.590073 (1.651)		
	-24.448362 (1.700)	-24.462914 (2.247)		
σTZ0	-24.528307 (0.210)	-24.597428 (0.876)		
	-24.449773 (0.459)	-24.467912 (0.701)		
σQZ0	-24.528948 (0.050)	-24.599335 (0.202)		
	-24.450495 (0.092)	-24.469277 (0.187)		
aσDZ0	-24.528097 (1.288)	-24.593793 (3.843)	-24.528170 (1.185)	-24.598056 (3.188)
	-24.449244 (2.516)	-24.465474 (4.041)		
aσTZ0	-24.528731 (0.593)	-24.598451 (1.595)	-24.528787 (0.457)	-24.604537 (1.242)
	-24.450251 (0.908)	-24.468750 (1.341)		
aσQZ0	-24.528978 (0.073)	-24.599573 (0.343)	-24.529139 (-0.014)	-24.606172 (0.228)
	-24.450554 (0.150)	-24.469418 (0.280)		

TABLE S7: C and C^- energies (E_h) at the HF and CISD levels used in the basis set optimization for Carbon atom and ΔE (mE_h) = E(XZ) - E(σXZ) in parentheses.

	C (^{3}P,^{1}D and ^{1}S)		C^- (^{4}S)	
BS	HF	CISD	HF	CISD
σDZ0	-37.702967 (3.227)	-37.764608 (4.421)		
	-37.644872 (3.455)	-37.710377 (4.912)		
	-37.606406 (3.831)	-37.658299 (5.652)		
σTZ0	-37.704776 (0.753)	-37.781076 (1.604)		
	-37.646803 (0.768)	-37.732189 (1.826)		
	-37.608663 (0.744)	-37.679733 (1.769)		
σQZ0	-37.705769 (0.172)	-37.785309 (0.378)		
	-37.647878 (0.177)	-37.738119 (0.490)		
	-37.609982 (0.179)	-37.686366 (0.484)		
aσDZ0	-37.704392 (4.147)	-37.773880 (9.579)	-37.706942 (3.330)	-37.809108 (7.147)
	-37.646541 (3.847)	-37.720975 (9.861)		
	-37.608716 (3.884)	-37.669870 (8.751)		
aσTZ0	-37.705470 (1.367)	-37.783684 (3.286)	-37.708030 (1.006)	-37.821644 (2.170)
	-37.647607 (1.290)	-37.736032 (3.539)		
	-37.609804 (1.264)	-37.684413 (3.709)		
aσQZ0	-37.705849 (0.238)	-37.786092 (0.868)	-37.708621 (0.126)	-37.825094 (0.320)
	-37.647991 (0.217)	-37.739327 (0.944)		
	-37.610205 (0.205)	-37.687924 (1.032)		

TABLE S8: N and N^- energies (E_h) at the HF and CISD levels used in the basis set optimization for Nitrogen atom and ΔE (mE_h) = E(XZ) - E(σXZ) in parentheses.

	N(^{4}S,^{2}D and ^{2}P)		N^-(^{3}P)	
BS	HF	CISD	HF	CISD
σDZ0	-54.395857 (7.443)	-54.486627 (9.585)		
	-54.290282 (7.776)	-54.386871 (10.167)		
	-54.256536 (8.028)	-54.343963 (10.547)		
σTZ0	-54.399077 (1.719)	-54.513925 (2.967)		
	-54.294052 (1.766)	-54.421342 (3.145)		
	-54.260626 (1.773)	-54.377664 (3.055)		
σQZ0	-54.400580 (0.404)	-54.521166 (0.960)		
	-54.295710 (0.399)	-54.431204 (0.859)		
	-54.262435 (0.396)	-54.387971 (0.845)		
aσDZ0	-54.398301 (8.430)	-54.502038 (17.399)	-54.318884 (7.134)	-54.470083 (13.756)
	-54.293425 (7.911)	-54.404652 (17.786)		
	-54.260180 (7.930)	-54.362644 (17.780)		
aσTZ0	-54.400171 (2.408)	-54.518324 (5.526)	-54.320504 (1.696)	-54.493960 (3.402)
	-54.295225 (2.278)	-54.427612 (5.926)		
	-54.262014 (2.255)	-54.384487 (6.037)		
aσQZ0	-54.400705 (0.480)	-54.522452 (1.473)	-54.321616 (0.312)	-54.501853 (1.031)
	-54.295923 (0.433)	-54.433208 (1.625)		
	-54.262723 (0.417)	-54.390283 (1.663)		

TABLE S9: O and O^- energies (E_h) at the HF and CISD levels used in the basis set optimization for Oxygen atom and ΔE (mE_h) = E(XZ) - E(σXZ) in parentheses.

	O(^{3}P,^{1}D and ^{1}S)		O^-(^{2}P)	
BS	HF	CISD	HF	CISD
σDZ0	-74.800818 (14.630)	-74.925871 (18.834)		
	-74.720269 (14.908)	-74.847967 (19.636)		
	-74.654996 (15.341)	-74.770459 (20.454)		
σTZ0	-74.806543 (3.465)	-74.972947 (5.479)		
	-74.726261 (3.512)	-74.898506 (5.752)		
	-74.661357 (3.546)	-74.818087 (5.821)		
σQZ0	-74.808762 (0.786)	-74.987134 (1.300)		
	-74.728546 (0.766)	-74.914150 (1.347)		
	-74.663798 (0.732)	-74.833981 (1.380)		
aσDZ0	-74.805708 (16.656)	-74.952428 (31.544)	-74.784447 (13.517)	-74.979927 (24.465)
	-74.725580 (16.251)	-74.874985 (31.861)		
	-74.660957 (16.265)	-74.797783 (32.223)		
aσTZ0	-74.808186 (4.586)	-74.982058 (10.484)	-74.787852 (3.529)	-75.016674 (7.748)
	-74.728048 (4.490)	-74.908398 (10.858)		
	-74.663427 (4.467)	-74.828211 (11.075)		
aσQZ0	-74.809070 (0.992)	-74.990432 (3.220)	-74.789241 (0.693)	-75.028664 (2.259)
	-74.728928 (0.959)	-74.917858 (3.408)		
	-74.664308 (0.938)	-74.837911 (3.530)		

TABLE S10: F and F^- energies (E_h) at the HF and CISD levels used in the basis set optimization for Fluorine atom and ΔE (mE_h) = E(XZ) - E(σXZ) in parentheses.

	$F(^2P)$		$F^-(^1S)$	
BS	HF	CISD	HF	CISD
σDZ0	-99.396065 (24.985)	-99.556194 (32.698)		
σTZ0	-99.405123 (5.929)	-99.620723 (9.019)		
σQZ0	-99.408477 (1.497)	-99.642618 (2.725)		
aσDZ0	-99.403887 (29.929)	-99.592525 (48.849)	-99.452462 (24.180)	-99.694843 (41.900)
aσTZ0	-99.407575 (7.471)	-99.633981 (15.994)	-99.457170 (6.363)	-99.741198 (13.348)
aσQZ0	-99.408857 (1.698)	-99.647332 (5.233)	-99.458861 (1.399)	-99.757950 (4.255)

TABLE S11: Ne and Ne_2 energies (E_h) at the HF and CISD levels used in the basis set optimization for Neon atom and ΔE (mE_h) = E(XZ) - E(σXZ) in parentheses.

	Ne (^{1}S, ^{3}P)		Ne_2($^1\Sigma_g^+$ 5.86 au)	
	HF	CISD	HF	CISD
σDZ0	-128.527973 (39.197)	-128.724929 (51.312)		
σTZ0	-128.541278 (9.417)	-128.805562 (13.646)		
σQZ0	-128.545805 (2.335)	-128.835189 (4.132)		
aσDZ0	-128.541267 (44.917)	128.781002 (79.533)	-257.082495 (89.792)	257.548966 (158.041)
aσTZ0	-128.545266 (11.993)	128.825897 (25.214)	-257.090464 (23.911)	257.635144 (50.012)
aσQZ0	-128.546646 (2.890)	128.842695 (8.419)	-257.093212 (5.694)	257.667499 (17.767)

TABLE S12: Na, Na^- and Na_2 energies (E_h) at the HF and CISD levels used in the basis set optimization for Sodium atom and ΔE (mE_h) = E(XZ) - E(σXZ) in parentheses.

	Na(^{2}S, ^{2}P)	Na$^-$(^{1}S)		Na$_2$($^1\Sigma_g^+$ 6.0 au)	
	HF	HF	CISD	HF	CISD
σDZ0	-161.855399 (2.371)			-323.709773 (5.008)	-323.736307 (5.184)
	-161.782692 (2.330)				
σTZ0	-161.858368 (0.372)			-323.716003 (0.787)	-323.743012 (0.617)
	-161.785535 (0.070)				
σQZ0	-161.858633 (-0.043)			-323.716645 (0.008)	-323.743922 (-0.014)
	-161.786134 (-0.033)				
$a\sigma$DZ0	-161.855890 (2.850)	-161.851689 (2.801)	-161.875702 (3.338)	-323.710964 (6.014)	-323.737409 (6.082)
	-161.783359 (2.948)				
aσTZ0	-161.858641 (0.642)	-161.854773 (0.602)	-161.878575 (0.601)	-323.716586 (1.344)	-323.743754 (1.314)
	-161.786011 (0.542)				
aσQZ0	-161.858759 (0.082)	-161.854906 (0.021)	-161.878722 (0.018)	-323.716974 (0.329)	-323.744254 (0.301)
	-161.786133 (-0.034)				

TABLE S13: Mg and Mg_2 energies (E_h) at the HF and CISD levels used in the basis set optimization for Magnesium atom and ΔE (mE_h) = E(XZ) - E(σXZ) in parentheses.

	Mg (^{1}S, ^{3}P)		Mg_2($^1\Sigma_g^+$ 6.8 au)	
	HF	CISD	HF	CISD
σDZ0	-199.610977 (2.680)	-199.643953 (2.779)		
	-199.542732 (2.749)	-199.548451 (4.329)		
σTZ0	-199.614138 (0.790)	-199.647796 (0.718)		
	-199.546000 (0.616)	-199.552340 (1.252)		
σQZ0	-199.614270 (0.037)	-199.648230 (0.077)		
	-199.546340 (0.039)	-199.552802 (0.265)		
aσDZ0	-199.613288 (4.949)	-199.646790 (5.499)	-399.221838 (9.862)	-399.294058 (11.059)
	-199.545092 (4.996)	-199.551233 (5.098)		
aσTZ0	-199.614355 (1.004)	-199.648223 (1.121)	-399.224105 (2.061)	-399.297972 (2.235)
	-199.546323 (0.924)	-199.552764 (0.961)		
aσQZ0	-199.614526 (0.292)	-199.648502 (0.339)	-399.224403 (0.586)	-399.298753 (0.678)
	-199.546582 (0.280)	-199.553070 (0.303)		

TABLE S14: Al and Al^- energies (E_h) at the HF and CISD levels used in the basis set optimization for Aluminum atom and ΔE (mE_h) = E(XZ) - E(σXZ) in parentheses.

	Al (^2P, ^4P)		Al^- (^3P)	
	HF	CISD	HF	CISD
σDZ0	-241.872284 (2.177)	-241.924044 (2.646)		
	-241.785983 (2.713)	-241.799333 (2.919)		
σTZ0	-241.875477 (0.447)	-241.932063 (0.833)		
	-241.789444 (0.996)	-241.805665 (1.059)		
σQZ0	-241.876421 (0.054)	-241.933941 (0.292)		
	-241.790571 (0.115)	-241.807265 (0.196)		
aσDZ0	-241.872922 (2.492)	-241.927428 (4.638)	-241.877350 (1.446)	-241.937171 (3.265)
	-241.786942 (3.588)	-241.801807 (4.372)		
aσTZ0	-241.876128 (0.959)	-241.933144 (1.619)	-241.877106 (0.367)	-241.944849 (1.030)
	-241.790237 (1.773)	-241.806731 (1.939)		
aσQZ0	-241.876437 (0.061)	-241.934099 (0.382)	-241.877780 (-0.136)	-241.946336 (0.132)
	-241.790597 (0.138)	-241.807371 (0.260)		

TABLE S15: Si and Si^- energies (E_h) at the HF and CISD levels used in the basis set optimization for Silicon atom and ΔE (mE_h) = E(XZ) - E(σXZ) in parentheses.

	Si (^{3}P,^{1}D and ^{1}S)		Si^-(^{4}S)	
BS	HF	CISD	HF	CISD
σDZ0	-288.849124 (2.726)	-288.917441 (3.087)		
	-288.809553 (3.302)	-288.880568 (3.404)		
	-288.751296 (3.625)	-288.837638 (3.800)		
σTZ0	-288.853247 (1.211)	-288.932457 (1.600)		
	-288.813953 (1.903)	-288.902394 (1.967)		
	-288.757103 (2.353)	-288.861116 (2.539)		
σQZ0	-288.854030 (0.054)	-288.935240 (0.265)		
	-288.814813 (0.677)	-288.906645 (0.375)		
	-288.758316 (-0.002)	-288.865641 (0.305)		
aσDZ0	-288.851539 (4.591)	-288.925417 (8.183)	-288.885834 (3.278)	-288.967556 (5.867)
	-288.812354 (4.961)	-288.889999 (8.164)		
	-288.756097 (4.400)	-288.841905 (7.565)		
aσTZ0	-288.853903 (1.676)	-288.934191 (2.742)	-288.887880 (0.206)	-288.978846 (0.935)
	-288.814753 (2.203)	-288.905129 (2.769)		
	-288.758562 (1.483)	-288.864137 (2.888)		
aσQZ0	-288.854117 (0.130)	-288.935775 (0.648)	-288.889176 (-0.125)	-288.982083 (0.204)
	-288.814966 (0.794)	-288.907453 (0.687)		
	-288.758846 (0.144)	-288.866708 (0.772)		

TABLE S16: P and P^- energies (E_h) at the HF and CISD levels used in the basis set optimization for Phosporus atom and ΔE (mE_h) = E(XZ) - E(σXZ) in parentheses.

	P(^{4}S,^{2}D and ^{2}P)		P^-(^{3}P)	
BS	HF	CISD	HF	CISD
σDZ0	-340.710985 (1.971)	-340.791874 (2.426)		
	-340.640515 (2.353)	-340.726536 (2.836)		
	-340.615768 (2.504)	-340.693489 (2.979)		
σTZ0	-340.717240 (1.256)	-340.817006 (1.547)		
	-340.647105 (1.279)	-340.760252 (1.715)		
	-340.622725 (1.303)	-340.727234 (1.722)		
σQZ0	-340.718423 (0.088)	-340.821866 (0.375)		
	-340.648434 (0.067)	-340.767477 (0.416)		
	-340.624153 (0.058)	-340.734735 (0.361)		
aσDZ0	-340.715778 (5.964)	-340.805295 (11.258)	-340.694818 (4.406)	-340.808503 (7.750)
	-340.645824 (5.711)	-340.742137 (11.421)		
	-340.621527 (5.757)	-340.710525 (11.169)		
aσTZ0	-340.717968 (1.844)	-340.819524 (3.157)	-340.697901 (1.501)	-340.833763 (2.533)
	-340.648057 (1.756)	-340.764234 (3.286)		
	-340.602383 (1.741)	-340.731379 (3.326)		
aσQZ0	-340.718515 (0.165)	-340.822749 (0.988)	-340.698547 (0.058)	-340.839582 (0.631)
	-340.648601 (0.161)	-340.768852 (1.060)		
	-340.624369 (0.163)	-340.736292 (1.132)		

TABLE S17: S and S^- energies (E_h) at the HF and CISD levels used in the basis set optimization for Sulfur atom and ΔE (mE_h) = E(XZ) - E(σXZ) in parentheses.

	S(^{3}P,^{1}D and ^{1}S)		S^-(^{2}P)	
BS	HF	CISD	HF	CISD
σDZ0	-397.496954 (4.637)	-397.601916 (5.325)		
	-397.444037 (4.694)	-397.552786 (5.473)		
	-397.399610 (4.776)	-397.497498 (5.391)		
σTZ0	-397.503353 (1.769)	-397.645877 (2.289)		
	-397.450708 (1.750)	-397.601355 (2.304)		
	-397.406745 (1.727)	-397.543908 (2.286)		
σQZ	-397.504459 (0.109)	-397.655112 (0.479)		
	-397.451874 (0.091)	-397.613211 (0.468)		
	-397.408005 (0.070)	-397.556037 (0.436)		
aσDZ	-397.501650 (8.229)	-397.621793 (16.882)	-397.533272 (5.544)	-397.676732 (11.621)
	-397.449112 (8.029)	-397.573674 (17.010)		
	-397.405262 (8.029)	-397.519944 (16.707)		
aσTZ	-397.504229 (2.500)	-397.651017 (5.275)	-397.536361 (0.929)	-397.712393 (2.381)
	-397.451697 (2.472)	-397.607295 (5.427)		
	-397.407887 (2.457)	-397.549686 (5.549)		
aσQZ0	-397.504634 (0.264)	-397.658156 (1.837)	-397.537821 (-0.123)	-397.724372 (1.094)
	-397.452093 (0.261)	-397.615552 (1.922)		
	-397.408280 (0.262)	-397.558624 (2.096)		

TABLE S18: Cl and Cl^{-} energies (E_h) at the HF and CISD levels used in the basis set optimization for Chlorine atom and ΔE (mE_h) = E(XZ) - E(σXZ) in parentheses.

	$Cl(^2P)$		$Cl^{-}(^1S)$	
BS	HF	CISD	HF	CISD
σDZ0	-459.474326 (7.494)	-459.602956 (9.341)		
σTZ0	-459.480363 (2.114)	-459.661637 (2.969)		
σQZ0	-459.481638 (0.207)	-459.678188 (0.677)		
aσDZ0	-459.477944 (9.993)	-459.627234 (22.478)	-459.571789 (8.144)	-459.744739 (18.399)
aσTZ0	-459.481223 (2.795)	-459.668991 (6.921)	-459.575638 (2.157)	-459.789863 (5.533)
aσQZ0	-459.481759 (0.306)	-459.681430 (2.768)	-459.576417 (0.064)	-459.806210 (2.146)

TABLE S19: Ar and Ar_2 energies (E_h) at the HF and CISD levels used in the basis set optimization for Argon atom and ΔE (mE_h) = E(XZ) - E(σXZ) in parentheses.

	Ar(^{1}S)		Ar_2 ($^1\Sigma_g^+$ 7.1 au)	
BS	HF	CISD	HF	CISD
σDZ0	-526.808740 (8.875)	-526.959778 (12.702)		
σTZ0	-526.815571 (2.437)	-527.031603 (3.859)		
σQZ0	-526.817001 (0.221)	-527.055026 (0.866)		
aσDZ0	-526.813642 (12.669)	-526.994191 (32.850)	-1053.626907 (25.319)	-1053.970483 (62.889)
aσTZ0	-526.816686 (3.334)	-527.041600 (9.429)	-1053.632978 (6.667)	-1054.058068 (17.742)
aσQZ0	-526.817210 (0.405)	-527.059969 (4.066)	-1053.633992 (0.802)	-1054.0920670 (7.373)

TABLE S20: HF and CISD average energy diferences XZ and σXZ basis sets.

		ΔE/mE_h					ΔE/mE_h		
Species	Method	σDZ0	σTZ0	σQZ0	Species	Method	aσDZ0	aσTZ0	aσQZ0
H / H_2	HF	2.1	0.2	0.1	H / H_2 / H^-	HF	1.7	0.1	0.0
	CISD	4.6	0.5	0.2		CISD	3.1	0.4	0.2
He	HF	4.7	0.0	0.0	He /He_2	HF	8.1	0.4	0.1
	CISD	5.7	0.4	0.1		CISD	14.1	2.2	0.7
Li/Li_2	HF	0.2	-0.1	0.0	Li/Li_2/Li^-	HF	0.3	-0.1	0.0
	CISD	0.6	0.1	0.0		CISD	0.9	0.1	0.0
Be	HF	0.2	-0.1	0.0	Be /Be_2	HF	0.9	0.2	0.1
	CISD	0.8	0.1	0.2		CISD	1.8	0.5	0.1
B	HF	1.3	0.3	0.1	B / B^-	HF	1.7	0.7	0.1
	CISD	1.9	0.8	0.2		CISD	3.7	1.4	0.3
C	HF	3.5	0.8	0.2	C / C^-	HF	3.9	1.3	0.2
	CISD	5.0	1.7	0.5		CISD	9.2	3.6	1.0
N	HF	7.7	1.8	0.4	N / N^-	HF	8.1	2.3	0.4
	CISD	10.1	3.1	0.8		CISD	17.7	5.9	1.6
O	HF	15.0	3.5	0.7	O / O^-	HF	16.4	4.5	1.0
	CISD	19.6	5.7	1.3		CISD	32.0	10.9	3.4
F	HF	25.0	5.9	1.5	F / F^-	HF	20.1	6.9	1.5
	CISD	32.7	9.0	2.7		CISD	45.2	14.7	4.7
Ne	HF	39.2	9.4	2.3	Ne / Ne_2	HF	44.9	12.0	2.9
	CISD	51.3	13.6	4.1		CISD	79.3	25.1	8.4
Na/Na_2	HF	3.2	0.4	0.0	Na/Na_2/Na^-	HF	4.7	1.0	0.1
	CISD	5.1	0.6	0.0		CISD	4.9	1.0	0.2
Mg	HF	2.7	0.7	0.0	Mg /Mg_2	HF	6.6	1.3	0.4
	CISD	3.6	1.0	0.2		CISD	7.2	1.4	0.4
Al	HF	2.4	0.7	0.1	Al / Al^-	HF	2.5	1.0	0.0
	CISD	2.8	0.9	0.2		CISD	4.1	1.5	0.3
Si	HF	3.2	1.8	0.2	Si / Si^-	HF	4.6	1.7	0.3
	CISD	3.4	2.0	0.3		CISD	7.9	2.8	0.7
P	HF	2.3	1.3	0.1	P / P^-	HF	5.8	1.8	0.2
	CISD	2.7	1.7	0.4		CISD	11.3	3.3	1.1
S	HF	4.7	1.7	0.1	S / S^-	HF	8.1	2.5	0.3
	CISD	5.7	2.3	0.5		CISD	16.8	5.4	2.0
Cl	HF	7.5	2.1	0.2	Cl / Cl^-	HF	9.1	2.5	0.2
	CISD	9.3	3.0	0.7		CISD	20.4	6.2	2.5
Ar	HF	8.9	2.4	0.2	Ar / Ar_2	HF	12.7	3.3	0.4
	CISD	12.7	3.9	0.9		CISD	32.1	9.2	3.9

TABLE S21: Differences in the average relative equilibrium distances are shown using the Dunning a5Z BS as a reference, along with the reaction energies in kJ/mol. The upper part shows each reactive system and its corresponding reaction energy value in kJ/mol using the a5Z BS

$1/2\ H_2 + 1/2\ F_2 \rightarrow HF$					-282.421
DZ0	2.061	-254.978	aDZ0	2.070	-281.797
TZ0	0.160	-273.399	aTZ0	0.361	-279.452
QZ0	0.096	-280.479	aQZ0	0.079	-282.456
σDZ0	1.770	-261.284	aσDZ0	1.113	-272.030
σTZ0	0.404	-277.055	aσTZ0	0.257	-280.503
σQZ0	0.132	-281.148	aσQZ0	0.071	-281.975
$1/2\ H_2 + 1/2\ Cl_2 \rightarrow HCl$					-96.562
DZ0	2.236	-106.637	aDZ0	2.464	-110.778
TZ0	0.475	-99.076	aTZ0	0.558	-99.908
QZ0	0.184	-98.146	aQZ0	0.212	-98.066
σDZ0	1.668	-102.887	aσDZ0	1.397	-107.511
σTZ0	0.480	-99.363	aσTZ0	0.372	-99.861
σQZ0	0.112	-98.030	aσQZ0	0.081	-97.797
$1/2\ H_2 + 1/2\ Li_2 \rightarrow HLi$					38.279
DZ0	1.492	46.522	aDZ0	1.610	39.758
TZ0	0.084	40.478	aTZ0	0.142	38.880
QZ0	0.028	38.902	aQZ0	0.029	38.396
σDZ0	0.639	51.086	aσDZ0	0.553	41.796
σTZ0	0.128	41.393	aσTZ0	0.063	39.047
σQZ0	0.030	38.942	aσQZ0	0.015	38.455
$1/2\ H_2 + 1/2\ Na_2 \rightarrow HNa$					74.422
DZ0	1.174	85.119	aDZ0	1.272	74.365
TZ0	0.058	78.340	aTZ0	0.138	76.094
QZ0	0.052	76.322	aQZ0	0.062	75.371
σDZ0	0.354	89.468	aσDZ0	0.282	78.746
σTZ0	0.096	79.542	aσTZ0	0.121	76.177
σQZ0	0.030	75.720	aσQZ0	0.034	74.244
$1/2\ Li_2 + 1/2\ F_2 \rightarrow LiF$					-446.344
DZ0	1.730	-410.000	aDZ0	1.801	-436.699
TZ0	0.281	-430.424	aTZ0	0.404	-437.859
QZ0	0.117	-443.437	aQZ0	0.068	-445.296
σDZ0	2.349	-398.858	aσDZ0	1.222	-428.563
σTZ0	0.579	-435.368	aσTZ0	0.179	-439.670
σQZ0	0.190	-444.878	aσQZ0	0.048	-445.176

	$1/2\ Na_2 + 1/2\ F_2 \rightarrow NaF$				-362.415
DZ0	2.349	-316.166	aDZ0	1.267	-358.020
TZ0	0.309	-341.159	aTZ0	0.270	-354.714
QZ0	0.084	-354.511	aQZ0	0.118	-359.562
σDZ0	2.483	-308.037	aσDZ0	0.689	-345.312
σTZ0	0.533	-339.412	aσTZ0	0.195	-354.359
σQZ0	0.228	-355.850	aσQZ0	0.095	-361.278
	$1/2\ Li_2 + 1/2\ Cl_2 \rightarrow LiCl$				-299.990
DZ0	2.154	-283.659	aDZ0	2.292	-298.407
TZ0	0.469	-294.066	aTZ0	0.593	-296.187
QZ0	0.157	-299.868	aQZ0	0.206	-300.583
σDZ0	2.199	-289.488	aσDZ0	1.839	-297.447
σTZ0	0.473	-295.000	aσTZ0	0.426	-294.898
σQZ0	0.160	-299.621	aσQZ0	0.075	-299.299
	$1/2\ Na_2 + 1/2\ Cl_2 \rightarrow NaCl$				-256.830
DZ0	1.358	-239.055	aDZ0	1.782	-260.090
TZ0	0.462	-247.357	aTZ0	0.601	-252.061
QZ0	0.185	-254.893	aQZ0	0.227	-256.758
σDZ0	1.509	-242.375	aσDZ0	1.353	-254.629
σTZ0	0.476	-247.809	aσTZ0	0.425	-251.113
σQZ0	0.153	-254.432	aσQZ0	0.064	-256.275
	$1/2\ C_2 + 1/2\ O_2 \rightarrow CO$				-587.566
DZ0	1.434	-584.112	aDZ0	1.613	-579.145
TZ0	0.483	-586.273	aTZ0	0.494	-583.399
QZ0	0.076	-588.462	aQZ0	0.090	-586.716
σDZ0	1.879	-587.293	aσDZ0	1.065	-586.785
σTZ0	0.567	-586.849	aσTZ0	0.288	-585.344
σQZ0	0.154	-587.481	aσQZ0	0.040	-587.187
	$1/2\ C_2 + 1/2\ S_2 \rightarrow CS$				-230.504
DZ0	2.159	-236.591	aDZ0	2.289	-239.631
TZ0	0.781	-230.064	aTZ0	0.754	-230.893
QZ0	0.243	-229.696	aQZ0	0.241	-230.235
σDZ0	2.262	-229.461	aσDZ0	1.220	-242.352
σTZ0	0.816	-230.703	aσTZ0	0.284	-232.607
σQZ0	0.203	-230.056	aσQZ0	0.041	-231.196

	$1/2\ Si_2 + 1/2\ O_2 \rightarrow SiO$				-484.950
DZ0	1.989	-429.039	aDZ0	2.281	-448.859
TZ0	0.705	-468.227	aTZ0	0.792	-472.704
QZ0	0.205	-478.785	aQZ0	0.223	-480.313
σDZ0	2.529	-441.454	aσDZ0	1.513	-474.154
σTZ0	0.831	-468.735	aσTZ0	0.566	-476.341
σQZ0	0.231	-479.951	aσQZ0	0.081	-483.838
	$1/2\ Si_2 + 1/2\ S_2 \rightarrow SiS$				-307.832
DZ0	2.249	-305.293	aDZ0	2.484	-306.796
TZ0	0.953	-304.122	aTZ0	0.990	-303.435
QZ0	0.303	-307.080	aQZ0	0.312	-306.903
σDZ0	2.514	-304.571	aσDZ0	1.519	-312.504
σTZ0	0.982	-304.074	aσTZ0	0.456	-303.943
σQZ0	0.242	-306.738	aσQZ0	0.065	-307.366
	$1/2\ B_2 + 1/2\ F_2 \rightarrow BF$				-575.940
DZ0	2.603	-548.055	aDZ0	2.878	-542.935
TZ0	0.393	-574.256	aTZ0	0.537	-569.838
QZ0	0.071	-576.829	aQZ0	0.115	-575.184
σDZ0	3.259	-548.261	aσDZ0	1.580	-562.500
σTZ0	0.651	-574.996	aσTZ0	0.409	-571.918
σQZ0	0.147	-577.158	aσQZ0	0.080	-574.780
	$1/2\ B_2 + 1/2\ Cl_2 \rightarrow BCl$				-285.565
DZ0	2.225	-300.172	aDZ0	2.602	-294.817
TZ0	0.702	-288.706	aTZ0	0.763	-286.109
QZ0	0.212	-287.087	aQZ0	0.242	-286.169
σDZ0	2.626	-297.771	aσDZ0	1.765	-293.387
σTZ0	0.770	-288.956	aσTZ0	0.432	-285.989
σQZ0	0.174	-287.200	aσQZ0	0.075	-285.387
	$1/2\ Al_2 + 1/2\ F_2 \rightarrow AlF$				-556.450
DZ0	2.963	-510.248	aDZ0	2.721	-542.212
TZ0	0.622	-537.823	aTZ0	0.766	-543.725
QZ0	0.164	-552.848	aQZ0	0.209	-553.737
σDZ0	3.614	-502.363	aσDZ0	1.793	-539.683
σTZ0	0.687	-542.053	aσTZ0	0.591	-548.137
σQZ0	0.130	-554.094	aσQZ0	0.082	-555.402

$1/2\ Al_2 + 1/2\ Cl_2 \rightarrow AlCl$					-341.682
DZ0	2.590	-334.484	aDZ0	3.232	-338.957
TZ0	0.861	-337.795	aTZ0	1.009	-337.274
QZ0	0.267	-341.384	aQZ0	0.314	-341.421
σDZ0	3.081	-336.129	aσDZ0	2.331	-345.072
σTZ0	0.834	-338.706	aσTZ0	0.680	-337.433
σQZ0	0.148	-341.484	aσQZ0	0.099	-341.202
$1/2\ H_2 + 1/2\ B_2 \rightarrow HB$					-22.255
DZ0	2.258	-28.508	aDZ0	2.289	-30.479
TZ0	0.293	-24.458	aTZ0	0.316	-24.231
QZ0	0.052	-22.994	aQZ0	0.068	-22.797
σDZ0	1.308	-23.697	aσDZ0	0.669	-27.474
σTZ0	0.274	-23.563	aσTZ0	0.161	-23.881
σQZ0	0.064	-22.769	aσQZ0	0.026	-22.683
$1/2\ H_2 + 1/2\ N_2 \rightarrow HN$					535.069
DZ0	2.010	534.805	aDZ0	1.989	517.966
TZ0	0.222	534.319	aTZ0	0.268	528.365
QZ0	0.039	535.407	aQZ0	0.060	533.480
σDZ0	0.955	529.338	aσDZ0	0.544	528.900
σTZ0	0.211	533.658	aσTZ0	0.143	529.788
σQZ0	0.051	534.188	aσQZ0	0.027	533.825
$1/2\ H_2 + 1/2\ P_2 \rightarrow HP$					274.009
DZ0	1.958	265.650	aDZ0	2.089	261.522
TZ0	0.423	268.534	aTZ0	0.454	266.031
QZ0	0.127	271.629	aQZ0	0.144	271.327
σDZ0	1.215	269.528	aσDZ0	0.738	265.869
σTZ0	0.364	268.606	aσTZ0	0.271	267.123
σQZ0	0.081	272.034	aσQZ0	0.019	272.032
$1/2\ H_2 + 1/2\ Al_2 \rightarrow HAl$					-34.236
DZ0	2.037	-36.719	aDZ0	2.309	-39.086
TZ0	0.427	-34.575	aTZ0	0.507	-34.488
QZ0	0.097	-34.396	aQZ0	0.123	-34.247
σDZ0	1.433	-30.321	aσDZ0	1.076	-37.455
σTZ0	0.318	-33.388	aσTZ0	0.340	-34.771
σQZ0	0.059	-34.218	aσQZ0	0.038	-34.608

$1/2\ Cl_2 + 1/2\ F_2 \rightarrow ClF$					-56.656
DZ0	3.436	-36.446	aDZ0	3.189	-62.879
TZ0	0.802	-48.454	aTZ0	0.919	-55.150
QZ0	0.314	-54.010	aQZ0	0.330	-56.451
σDZ0	4.416	-33.525	aσDZ0	2.397	-54.454
σTZ0	0.990	-49.522	aσTZ0	0.702	-57.099
σQZ0	0.201	-54.460	aσQZ0	0.121	-57.047
$1/2\ P_2 + 1/2\ N_2 \rightarrow PN$					110.363
DZ0	1.887	125.499	aDZ0	2.095	114.177
TZ0	0.661	115.531	aTZ0	0.691	112.790
QZ0	0.184	112.868	aQZ0	0.203	111.864
σDZ0	1.906	118.965	aσDZ0	1.051	107.950
σTZ0	0.732	115.158	aσTZ0	0.428	110.608
σQZ0	0.172	110.833	aσQZ0	0.042	109.373
$2\ N_2 \rightarrow N_4$					753.173
DZ0	1.356	773.469	aDZ0	1.739	756.287
TZ0	0.401	756.410	aTZ0	0.439	750.041
QZ0	0.059	755.806	aQZ0	0.092	753.552
σDZ0	1.449	775.537	aσDZ0	1.156	799.984
σTZ0	0.444	757.785	aσTZ0	0.279	760.597
σQZ0	0.108	749.928	aσQZ0	0.054	755.618
$2\ P_2 \rightarrow P_4$					-234.607
DZ0	2.181	-136.055	aDZ0	2.482	-146.460
TZ0	0.871	-206.611	aTZ0	0.895	-210.938
QZ0	0.269	-222.655	aQZ0	0.296	-225.238
σDZ0	2.287	-134.940	aσDZ0	1.697	-152.993
σTZ0	0.936	-207.263	aσTZ0	0.575	-206.473
σQZ0	0.173	-226.158	aσQZ0	0.045	-229.383
$NO^- + O_2 \rightarrow NO_3^-$					-627.183
DZ0	1.572	-632.568	aDZ0	1.371	-607.904
TZ0	0.994	-645.863	aTZ0	0.597	-620.002
QZ0	0.630	-650.481	aQZ0	0.159	-627.254
σDZ0	2.451	-634.819	aσDZ0	1.602	-611.933
σTZ0	1.208	-656.607	aσTZ0	0.604	-630.177
σQZ0	0.783	-656.541	aσQZ0	0.229	-629.675

$PS^- + S_2 \rightarrow PS_3^-$					-541.580
DZ0	2.461	-499.532	aDZ0	2.600	-486.413
TZ0	1.156	-529.237	aTZ0	1.060	-515.932
QZ0	0.430	-540.816	aQZ0	0.361	-533.789
σDZ0	2.669	-498.455	aσDZ0	1.794	-512.204
σTZ0	1.172	-529.285	aσTZ0	0.520	-522.041
σQZ0	0.300	-546.682	aσQZ0	0.079	-539.124
$PO^- + O_2 \rightarrow PO_3^-$					-998.016
DZ0	2.964	-855.399	aDZ0	2.885	-864.892
TZ0	1.108	-974.899	aTZ0	0.919	-948.574
QZ0	0.479	-998.344	aQZ0	0.279	-980.402
σDZ0	3.328	-884.016	aσDZ0	1.832	-940.632
σTZ0	1.195	-978.042	aσTZ0	0.696	-969.394
σQZ0	0.454	-1012.470	aσQZ0	0.133	-998.212
$NS^- + S_2 \rightarrow NS_3^-$					-318.190
DZ0	3.274	-66.933	aDZ0	2.739	-63.381
TZ0	1.366	-351.539	aTZ0	1.060	-324.852
QZ0	0.581	-340.712	aQZ0	0.377	-322.285
σDZ0	3.474	-362.518	aσDZ0	1.892	-334.740
σTZ0	1.496	-354.820	aσTZ0	0.605	-327.821
σQZ0	0.527	-341.454	aσQZ0	0.167	-322.076
$NH_3 + HeH^+ \rightarrow NH_4^+ + He$					-689.240
DZ0	1.729	-725.999	aDZ0	1.013	-693.999
TZ0	0.305	-701.467	aTZ0	0.210	-689.609
QZ0	0.094	-693.775	aQZ0	0.043	-689.318
σDZ0	0.851	-727.253	aσDZ0	0.419	-697.366
σTZ0	0.331	-704.706	aσTZ0	0.071	-691.273
σQZ0	0.108	-695.432	aσQZ0	0.019	-689.818
$PH_3 + HeH^+ \rightarrow PH_4^+ + He$					-611.98
DZ0	1.069	-619.881	aDZ0	1.053	-612.366
TZ0	0.157	-611.974	aTZ0	0.209	-609.042
QZ0	0.041	-612.156	aQZ0	0.059	-611.123
σDZ0	0.563	-616.691	aσDZ0	0.255	-617.730
σTZ0	0.055	-615.322	aσTZ0	0.062	-611.244
σQZ0	0.020	-614.086	aσQZ0	0.005	-612.525

$NH_3 + NeH^+ \rightarrow NH_4^+ + Ne$					-663.821
DZ0	1.543	-687.347	aDZ0	1.353	-682.096
TZ0	0.321	-665.412	aTZ0	0.188	-662.367
QZ0	0.130	-664.309	aQZ0	0.050	-662.681
σDZ0	1.138	-702.444	aσDZ0	0.625	-672.985
σTZ0	0.315	-674.098	aσTZ0	0.084	-665.305
σQZ0	0.100	-668.018	aσQZ0	0.019	-664.090
$PH_3 + NeH^+ \rightarrow PH_4^+ + Ne$					-586.579
DZ0	0.882	-581.229	aDZ0	1.394	-600.462
TZ0	0.173	-575.919	aTZ0	0.186	-581.800
QZ0	0.077	-582.689	aQZ0	0.067	-584.486
σDZ0	0.851	-591.883	aσDZ0	0.461	-593.348
σTZ0	0.039	-584.714	aσTZ0	0.075	-585.276
σQZ0	0.013	-586.672	aσQZ0	0.005	-586.796
$NH_3 + ArH^+ \rightarrow NH_4^+ + Ar$					-493.004
DZ0	1.399	-518.718	aDZ0	0.899	-497.640
TZ0	0.270	-498.197	aTZ0	0.177	-489.413
QZ0	0.102	-496.181	aQZ0	0.061	-493.099
σDZ0	0.777	-530.147	aσDZ0	0.336	-501.330
σTZ0	0.391	-503.253	aσTZ0	0.154	-489.564
σQZ0	0.112	-497.056	aσQZ0	0.040	-493.055
$PH_3 + ArH^+ \rightarrow PH_4^+ + Ar$					-415.762
DZ0	0.738	-412.600	aDZ0	0.939	-416.007
TZ0	0.123	-408.703	aTZ0	0.175	-408.846
QZ0	0.050	-414.561	aQZ0	0.078	-414.904
σDZ0	0.489	-419.585	aσDZ0	0.173	-421.694
σTZ0	0.115	-413.869	aσTZ0	0.145	-409.535
σQZ0	0.024	-415.710	aσQZ0	0.026	-415.762
$OH_3^+ + CN^- \rightarrow HCN + H_2O$					-773.455
DZ0	1.608	-809.423	aDZ0	1.125	-768.984
TZ0	0.304	-792.547	aTZ0	0.308	-773.821
QZ0	0.084	-783.345	aQZ0	0.073	-773.539
σDZ0	0.942	-820.494	aσDZ0	0.613	-777.413
σTZ0	0.370	-796.873	aσTZ0	0.146	-782.679
σQZ0	0.105	-789.776	aσQZ0	0.018	-774.979

$SH_3^+ + CP^- \rightarrow HCP + H_2S$					-833.560
DZ0	1.041	-871.886	aDZ0	1.129	-817.545
TZ0	0.259	-859.582	aTZ0	0.301	-831.937
QZ0	0.082	-846.526	aQZ0	0.094	-833.624
σDZ0	0.895	-881.791	aσDZ0	0.455	-836.420
σTZ0	0.247	-863.245	aσTZ0	0.157	-835.878
σQZ0	0.068	-850.140	aσQZ0	0.022	-834.317
$OH_3^+ + CP^- \rightarrow HCP + H_2O$					-845.615
DZ0	1.635	-862.546	aDZ0	1.163	-835.626
TZ0	0.400	-860.434	aTZ0	0.393	-843.756
QZ0	0.122	-853.262	aQZ0	0.102	-844.817
σDZ0	1.019	-867.693	aσDZ0	0.700	-855.016
σTZ0	0.444	-862.000	aσTZ0	0.213	-848.882
σQZ0	0.125	-856.481	aσQZ0	0.022	-846.374
$SH_3^+ + CN^- \rightarrow HCN + H_2S$					-761.400
DZ0	1.014	-818.764	aDZ0	1.090	-750.903
TZ0	0.163	-791.695	aTZ0	0.216	-762.002
QZ0	0.044	-776.609	aQZ0	0.065	-762.346
σDZ0	0.818	-834.593	aσDZ0	0.368	-758.816
σTZ0	0.173	-798.118	aσTZ0	0.090	-769.676
σQZ0	0.048	-783.434	aσQZ0	0.018	-762.922
$NH_4^+ + OH^- \rightarrow NH_3 + H_2O$					776.090
DZ0	1.729	934.566	aDZ0	0.794	770.390
TZ0	0.381	872.693	aTZ0	0.252	775.698
QZ0	0.128	835.114	aQZ0	0.050	776.358
σDZ0	0.981	944.831	aσDZ0	0.495	788.447
σTZ0	0.430	895.461	aσTZ0	0.120	783.041
σQZ0	0.155	848.531	aσQZ0	0.020	779.298
$NH_4^+ + SH^- \rightarrow NH_3 + H_2S$					602.964
DZ0	1.265	656.435	aDZ0	0.865	599.613
TZ0	0.268	630.703	aTZ0	0.213	603.503
QZ0	0.087	616.403	aQZ0	0.062	602.154
σDZ0	0.844	658.658	aσDZ0	0.308	616.089
σTZ0	0.248	639.604	aσTZ0	0.075	614.443
σQZ0	0.081	624.622	aσQZ0	0.020	606.192

$PH_4^+ + OH^- \rightarrow PH_3 + H_2O$					853.332
DZ0	1.289	1040.684	aDZ0	0.821	852.023
TZ0	0.282	962.187	aTZ0	0.251	856.265
QZ0	0.093	916.734	aQZ0	0.061	854.553
σDZ0	0.790	1055.393	aσDZ0	0.386	868.083
σTZ0	0.247	984.845	aσTZ0	0.114	863.071
σQZ0	0.097	929.877	aσQZ0	0.011	856.592

$PH_4^+ + SH^- \rightarrow PH_3 + H_2S$					680.206
DZ0	0.825	762.553	aDZ0	0.892	681.246
TZ0	0.170	720.197	aTZ0	0.212	684.070
QZ0	0.052	698.023	aQZ0	0.073	680.349
σDZ0	0.652	769.219	aσDZ0	0.199	695.725
σTZ0	0.064	728.988	aσTZ0	0.069	694.473
σQZ0	0.022	705.968	aσQZ0	0.010	683.486

$4\ LiOH + 2\ Cl_2 \rightarrow 4\ LiCl + O_2 + 2\ H_2O$					184.303
DZ0	1.575	247.143	aDZ0	1.501	236.968
TZ0	0.449	199.222	aTZ0	0.502	192.266
QZ0	0.174	190.488	aQZ0	0.132	190.435
σDZ0	1.471	253.770	aσDZ0	1.276	236.642
σTZ0	0.544	185.432	aσTZ0	0.310	180.455
σQZ0	0.213	182.062	aσQZ0	0.051	183.715

$4\ NaOH + 2\ Cl_2 \rightarrow 4\ NaCl + O_2 + 2\ H_2O$					350.219
DZ0	1.492	442.149	aDZ0	1.210	409.023
TZ0	0.489	386.975	aTZ0	0.521	358.036
QZ0	0.150	370.377	aQZ0	0.154	359.989
σDZ0	1.471	449.869	aσDZ0	1.083	408.272
σTZ0	0.483	366.894	aσTZ0	0.318	351.449
σQZ0	0.176	352.990	aσQZ0	0.054	349.285

$4\ LiOH + 2\ F_2 \rightarrow 4\ LiF + O_2 + 2\ H_2O$					769.717
DZ0	1.393	752.507	aDZ0	1.290	790.139
TZ0	0.369	744.653	aTZ0	0.421	758.952
QZ0	0.157	764.764	aQZ0	0.072	769.286
σDZ0	1.535	691.252	aσDZ0	1.012	761.107
σTZ0	0.590	746.906	aσTZ0	0.223	759.541
σQZ0	0.226	763.093	aσQZ0	0.039	767.223

4 NaOH + 2 F_2 → 4 NaF + O_2 + 2 H_2O					772.558
DZ0	1.920	750.593	aDZ0	0.989	800.742
TZ0	0.427	762.181	aTZ0	0.380	768.648
QZ0	0.132	768.851	aQZ0	0.107	771.204
σDZ0	1.892	712.517	aσDZ0	0.798	771.004
σTZ0	0.540	733.304	aσTZ0	0.235	764.431
σQZ0	0.250	758.661	aσQZ0	0.068	769.297
Be_2C + 4 HF → CH_4 + 2 BeF_2					-1570.763
DZ0	1.070	-1552.877	aDZ0	1.229	-1497.433
TZ0	0.217	-1582.241	aTZ0	0.351	-1560.173
QZ0	0.037	-1578.803	aQZ0	0.058	-1569.121
σDZ0	1.229	-1460.329	aσDZ0	0.775	-1583.795
σTZ0	0.289	-1584.642	aσTZ0	0.149	-1570.056
σQZ0	0.059	-1579.298	aσQZ0	0.032	-1572.089
Mg_2Si + 4 HCl → SiH_4 + 2 $MgCl_2$					-1082.695
DZ0	1.000	-1010.147	aDZ0	1.388	-1015.550
TZ0	0.149	-1049.114	aTZ0	0.328	-1047.719
QZ0	0.052	-1073.947	aQZ0	0.126	-1075.145
σDZ0	1.381	-982.483	aσDZ0	0.865	-1029.221
σTZ0	0.307	-1044.316	aσTZ0	0.247	-1049.033
σQZ0	0.062	-1072.347	aσQZ0	0.084	-1072.291
HCN → HNC					62.119
DZ0	1.161	66.321	aDZ0	1.145	61.098
TZ0	0.135	62.488	aTZ0	0.172	62.110
QZ0	0.017	62.049	aQZ0	0.039	61.867
σDZ0	0.797	68.687	aσDZ0	0.437	61.817
σTZ0	0.181	61.975	aσTZ0	0.084	63.028
σQZ0	0.047	62.527	aσQZ0	0.012	62.119
FCN → FNC					297.069
DZ0	1.134	287.983	aDZ0	1.413	286.019
TZ0	0.256	293.989	aTZ0	0.298	294.094
QZ0	0.029	296.611	aQZ0	0.057	296.131
σDZ0	1.185	295.287	aσDZ0	0.790	291.149
σTZ0	0.317	294.942	aσTZ0	0.168	295.390
σQZ0	0.075	296.034	aσQZ0	0.025	296.761

ClCN → ClNC					181.798
DZ0	1.348	188.078	aDZ0	1.407	183.242
TZ0	0.383	182.417	aTZ0	0.378	181.552
QZ0	0.114	182.594	aQZ0	0.125	181.686
σDZ0	1.436	189.514	aσDZ0	0.720	180.282
σTZ0	0.408	183.236	aσTZ0	0.218	180.789
σQZ0	0.078	182.410	aσQZ0	0.020	181.355
2 CH_4 → C_2H_2 + 3 H_2					-447.829
DZ0	1.870	-455.182	aDZ0	1.911	-460.930
TZ0	0.141	-450.379	aTZ0	0.176	-450.946
QZ0	0.033	-449.145	aQZ0	0.054	-448.586
σDZ0	0.870	-441.080	aσDZ0	0.600	-446.568
σTZ0	0.162	-448.844	aσTZ0	0.080	-451.015
σQZ0	0.025	-449.096	aσQZ0	0.016	-448.570
2 SiH_4 → Si_2H_2 + 3 H_2					-526.134
DZ0	1.461	-522.755	aDZ0	1.579	-521.457
TZ0	0.271	-517.117	aTZ0	0.310	-517.452
QZ0	0.071	-524.819	aQZ0	0.086	-524.488
σDZ0	0.874	-489.955	aσDZ0	0.592	-516.601
σTZ0	0.241	-515.481	aσTZ0	0.135	-525.127
σQZ0	0.066	-524.350	aσQZ0	0.027	-528.211
CO_2 + SiO → CO + SiO_2					100.285
DZ0	1.841	155.199	aDZ0	2.225	136.938
TZ0	0.539	113.290	aTZ0	0.639	111.335
QZ0	0.154	106.594	aQZ0	0.193	105.294
σDZ0	1.888	157.465	aσDZ0	1.158	114.651
σTZ0	0.628	114.381	aσTZ0	0.350	106.763
σQZ0	0.163	105.590	aσQZ0	0.051	101.780
CO_2 + SO → CO + SO_2					-105.452
DZ0	2.128	-1.481	aDZ0	2.479	-26.522
TZ0	0.739	-70.232	aTZ0	0.849	-71.756
QZ0	0.253	-88.796	aQZ0	0.299	-89.646
σDZ0	2.234	6.726	aσDZ0	1.239	-71.863
σTZ0	0.298	-107.534	aσTZ0	0.315	-96.224
σQZ0	0.187	-95.511	aσQZ0	0.024	-105.060

$SO_2 + SiO \rightarrow SO + SiO_2$					-205.737
DZ0	2.692	-156.679	aDZ0	3.153	-163.460
TZ0	0.858	-183.522	aTZ0	1.016	-183.091
QZ0	0.343	-195.389	aQZ0	0.390	-194.940
σDZ0	2.793	-150.739	aσDZ0	1.586	-186.513
σTZ0	0.467	-221.916	aσTZ0	0.446	-202.987
σQZ0	0.234	-201.101	aσQZ0	0.052	-206.840
$C_3H_3^+ \rightarrow C_2H_2 + CH^+$					-793.323
DZ0	1.639	-761.980	aDZ0	1.682	-757.784
TZ0	0.222	-782.983	aTZ0	0.243	-782.726
QZ0	0.042	-790.664	aQZ0	0.057	-791.059
σDZ0	1.061	-753.059	aσDZ0	0.561	-756.061
σTZ0	0.249	-779.818	aσTZ0	0.062	-783.342
σQZ0	0.054	-790.497	aσQZ0	0.009	-791.395
$Si_3H_3^+ \rightarrow Si_2H_2 + SiH^+$					-536.948
DZ0	1.329	-485.127	aDZ0	1.489	-484.104
TZ0	0.441	-518.960	aTZ0	0.468	-519.832
QZ0	0.124	-530.426	aQZ0	0.139	-531.317
σDZ0	1.342	-482.737	aσDZ0	0.746	-491.530
σTZ0	0.441	-516.394	aσTZ0	0.203	-522.077
σQZ0	0.122	-531.109	aσQZ0	0.022	-534.047
$CO_3^{2-} + CO \rightarrow C_2O_4^{2-}$					-233.921
DZ0	0.706	-265.764	aDZ0	0.886	-226.170
TZ0	0.229	-259.847	aTZ0	0.246	-233.109
QZ0	0.115	-253.953	aQZ0	0.050	-234.028
σDZ0	0.671	-272.984	aσDZ0	0.482	-227.314
σTZ0	0.300	-268.841	aσTZ0	0.172	-236.668
σQZ0	0.153	-263.455	aσQZ0	0.044	-236.932
$2/3\ O_3 + 1/3\ S_3 \rightarrow SO_2$					-443.220
DZ0	1.692	-304.040	aDZ0	1.920	-339.244
TZ0	0.699	-398.545	aTZ0	0.761	-402.234
QZ0	0.259	-423.927	aQZ0	0.264	-424.932
σDZ0	2.133	-323.364	aσDZ0	1.318	-402.236
σTZ0	0.269	-446.722	aσTZ0	0.315	-437.303
σQZ0	0.172	-433.227	aσQZ0	0.038	-444.701

TABLE S22: The statistical analysis (average error, maximum error and deviation) of differences in the average equilibrium distances and reaction energies using the Dunning a5Z BS as a reference.

Distance error %							
BS	Med	Max	desv	BS	Med	Max	desv
DZ	1.76	3.43	0.61	aDZ	1.76	3.23	0.68
TZ	0.45	1.36	0.29	aTZ	0.48	1.06	0.27
QZ	0.15	0.62	0.13	aQZ	0.14	0.39	0.099
σDZ0	1.66	4.41	0.90	aσDZ0	0.99	2.39	0.55
σTZ0	0.48	1.49	0.31	aσTZ0	0.28	0.70	0.18
σQZ0	0.15	0.78	0.12	aσQZ0	0.049	0.23	0.041
Energy error %							
BS	Med	Max	desv	BS	Med	Max	desv
DZ	13.09	98.59	18.24	aDZ	8.78	80.08	15.86
TZ	4.55	33.39	5.50	aTZ	2.61	31.95	4.70
QZ	2.07	15.79	2.85	aQZ	1.02	14.99	2.21
σDZ0	12.82	106.37	17.06	aσDZ0	5.29	34.79	7.50
σTZ0	3.92	15.41	4.14	aσTZ0	1.78	11.99	2.11
σQZ0	1.93	12.62	2.65	aσQZ0	0.42	2.22	0.46

TABLE S23: MP2 and B3LYP-D3 energies (in E_h) for the compounds of the reaction: $C_{16}H_{10} + C_{20}N_4H_{12} \rightarrow C_{36}N_4H_{22}$

	MP2: $C_{16}H_{10}$+ $C_{20}N_4H_{12} \rightarrow C_{36}N_4H_{22}$			
BS	#p/#c	$C_{16}H_{10}$	$C_{20}N_4H_{12}$	$C_{36}N_4H_{22}$
aDZ	1642/1118	-613.96798045	-982.03336165	-1599.63273659
aTZ	2870/2346	-614.48264497	-982.23044008	-1600.96188014
aQZ	4776/4212	-614.64787792	-982.28406249	-1601.39470539
def2-SVPD	1420/976	-613.47249931	-981.20739357	-1598.33276603
def2-TZVPPD	2498/1854	-614.46336771	-982.25358856	-1600.90649452
def2-QZVPPD	4112/3246	-614.63349630	-982.29100345	—[a]
aσDZ0	1784/1118	-614.17987906	-982.18058489	-1600.18054258
aσTZ0	3012/2346	-614.55168815	-982.27034498	-1601.13829585
aσQZ0	4918/4212	-614.66930725	-982.29144933	-1601.44154999
anoaDZR	2880/1118	-614.21637287	-982.23341632	-1600.28115771
anoaTZR	4050/2346	-614.55232870	-982.28404771	-1601.14401794
anoaDZN	3412/1118	-614.29564790	-982.23604946	-1600.46409609
anoaTZN	4972/2386	-614.58722170	-982.28828068	-1601.22508581
anoaQZN	6514/3900	-614.67525481	-982.29490867	-1601.45674036
	B3LYP-D3: $C_{16}H_{10}$+ $C_{20}N_4H_{12} \rightarrow C_{36}N_4H_{22}$			
BS	#p/#c	$C_{16}H_{10}$	$C_{20}N_4H_{12}$	$C_{36}N_4H_{22}$
aDZ	1642/1118	-615.44856844	-987.70318436	-1603.36216634
aTZ	2870/2346	-615.59160096	-987.92357506	-1603.72558027
aQZ	4776/4212	-615.63165010	-987.98809797	-1603.82978817
def2-SVPD	1420/976	-614.97352412	-986.92064866	-1602.11876995
def2-TZVPPD	2498/1854	-615.60490690	-987.94585147	-1603.74611218
def2-QZVPPD	4112/3246	-615.63884685	-987.99976656	—[a]
apc1	1602/1118	-615.29097505	-987.44811521	-1602.94997381
apc2	3052/2346	-615.60379700	-987.94269726	-1603.75664788
apc3	5588/4478	-615.64452914	—[a]	—[a]
aσDZ0	1784/1118	-615.54970121	-987.86300266	-1603.62020045
aσTZ0	3012/2346	-615.62234784	-987.97321569	-1603.80636285
aσQZ0	4918/4212	-615.64147875	-988.00362724	-1603.85543182
anoaDZR	2880/1118	-615.59021997	-987.92681819	-1603.72506706
anoaTZR	4050/2346	-615.62951040	-987.98575683	-1603.82670974
anoaDZN	3412/1118	-615.60046456	-987.94095876	-1603.74694106
anoaTZN	4972/2386	-615.63647012	-987.99595388	-1603.84283737
anoaQZN	6514/3900	-615.64492222	-988.00885213	-1603.86395632

[a] Not converged

TABLE S24: Lowest Overlap Eigenvalues (LOE) and Number of Eigenvalues Below 10^{-7} (NEBT) from Calculations on Pyrene, Porphin, and Tetra-benzene Porphyrin.

	$C_{16}H_{10}$			$C_{20}N_4H_{12}$			$C_{36}N_4H_{22}$		
BS	#p/#c	LOE	NEBT	#p/#c	LOE	NEBT	#p/#c	LOE	NEBT
aDZ	670/458	1.76(-7)	0	972/660	3.86(-7)	0	1642/1118	1.26(-7)	0
aTZ	1178/966	1.38(-7)	0	1692/1380	1.10(-7)	0	2870/2346	2.38(-9)	8
aQZ	1968/1740	1.89(-8)	7	2808/2472	9.62(-9)	9	4776/4212	4.09(-10)	38
def2-SVPD	580/400	3.29(-6)	0	840/576	6.45(-6)	0	1420/976	3.16(-6)	0
def2-TZVPPD	1022/762	1.31(-7)	0	1476/1092	1.41(-7)	0	2498/1854	2.24(-8)	4
def2-QZVPPD	1688/1338	6.5(-10)	13	2424/1908	1.35(-9)	12	4112/3246	1.96(-10)	35
apc1	654/458	1.56(-7)	0	948/660	1.65(-7)	0	1602/1118	3.23(-9)	4
apc2	1252/966	4.70(-10)	19	1800/1380	1.26(-9)	18	3052/2346	1.20(-10)	68
apc3	2300/1850	1.30(-11)	78	3288/2628	8.02(-12)	101	5588/4478	3.46(-13)	274
aσDZ0	728/458	1.71(-5)	0	1056/660	3.81(-5)	0	1784/1118	1.78(-5)	0
aσTZ0	1236/966	1.46(-6)	0	1776/1380	3.25(-6)	0	3012/2346	1.46(-6)	0
aσQZ0	2026/1740	2.93(-7)	0	2892/2472	2.69(-7)	0	4918/4212	1.51(-7)	0
anoaDZR	1176/458	5.64(-5)	0	1704/660	7.53(-5)	0	2880/1118	4.71(-5)	0
anoaTZR	1662/962	3.83(-7)	0	2388/1380	6.08(-7)	0	4050/2346	4.00(-7)	0
anoaDZN	1396/474	8.66(-5)	0	2016/684	1.31(-4)	0	3412/1118	9.33(-5)	0
anoaTZN	2044/982	7.41(-6)	0	2928/1404	1.35(-5)	0	4972/2386	8.98(-6)	0
anoaQZN	2686/1996	8.68(-7)	0	3828/2304	9.10(-7)	0	6514/3900	5.33(-7)	0

TABLE S25: Lowest overlap eigenvalues (LOE) of calculations for CB[4] and CB[6]

	CB[4] $C_{24}N_{16}O_8H_{24}$		
BS	#prim.	#cont.	LOE
aDZ	1984	1320	5.33(-7)
aTZ	3384	2760	1.02(-7)
aQZ	5616	4944	6.08(-9)
aσDZ0	2112	1320	8.67(-5)
aσTZ0	3552	2760	5.95(-6)
aσQZ0	5784	4944	5.34(-7)
	CB[6] $C_{36}N_{24}O_{12}H_{36}$		
BS	#prim.	#cont.	LOE
aDZ	2916	1980	3.25(-7)
aTZ	5076	4140	5.24(-8)
aQZ	8424	7416	5.01(-9)
aσDZ0	3168	1980	7.04(-5)
aσTZ0	5328	4140	2.63(-6)
aσQZ0	8676	7416	6.21(-7)

TABLE S26: B3LYP and MP2 calculations for ground states of 2 $P_2S_5 \rightarrow P_4S_{10}$: total energies (in Eh) and comparison of reaction energy(in kJ/mol) for conventional vs. RI calculations

BS	#prim.	#cont.	B3LYP-P_2S_5	B3LYP-P_4S_{10}	ΔE	ΔE_{RI}
aDZ	700	378	-2673.449751	-5346.962133	164.44	166.29
aTZ	1050	700	-2673.555753	-5347.173476	162.70	162.79
aQZ	1568	1176	-2673.587434	-5347.237697	164.94	164.96
a5Z	2268	1834	-2673.619307	-5347.303145	169.42	169.42
aσDZ0	742	378	-2673.530656	-5347.118102	149.10	150.45
aσTZ0	1092	700	-2673.581190	-5347.224313	162.61	162.63
aσQZ0	1610	1176	-2673.610022	-5347.286702	175.02	175.06
BS	#prim.	#cont.	MP2-P_2S_5	MP2-P_4S_{10}	ΔE	ΔE_{RI}
aDZ	700	378	-2670.146102	-5340.402608	289.87	291.17
aTZ	1050	700	-2670.489314	-5341.102825	326.08	326.02
aQZ	1568	1176	-2670.608100	-5341.343055	333.06	332.90
aσDZ0	742	378	-2670.270205	-5340.638688	258.03	258.98
aσTZ0	1092	700	-2670.533195	-5341.187831	318.84	318.90
aσQZ0	1610	1176	-2670.631004	-5341.389891	335.76	335.48

TABLE S27: RI-B3LYP-D3 and RI-MP2 calculations for Adenine, Thymine, Guanine, Cytosine and Uracil: total energy (in Eh), error RI (in kJ/mol) and distance average relative error for distances smaller than 3 Å.

	Adenine							
BS	#prim.	#cont.	RI-B3LYP+D3	er RI	%dis.Av.er.	RI-MP2	er RI	%dis.Av.er.
aDZ	324	220	-467.1385235	0.22	0.513	-466.094850	2.93	0.977
aTZ	564	460	-467.2453431	0.04	0.046	-466.484750	1.36	0.247
aQZ	936	824	-467.2775041	0.03	0.013	-466.613041	0.43	0.046
a5Z	1484	1336	-467.2867182	0.02		-466.658049	0.17	
aσDZ0	352	220	-467.2191384	0.14	0.197	-466.2610438	3.50	0.464
aσTZ0	592	460	-467.2699322	0.09	0.023	-466.5394397	1.43	0.136
aσQZ0	964	824	-467.2845703	0.03	0.004	-466.6295329	0.59	0.007
	Thymine							
BS	#prim.	#cont.	RI-B3LYP+D3	er RI	%dis.Av.er.	RI-MP2	er RI	%dis.Av.er.
aDZ	381	261	-453.9718356	0.19	0.503	-452.972759	2.51	0.999
aTZ	672	552	-454.0834244	0.03	0.043	-453.356869	1.27	0.185
aQZ	1125	996	-454.1157436	0.04	0.010	-453.483585	0.39	0.042
a5Z	1797	1623	-454.1157436	0.03		-453.528374	0.17	
aσDZ0	414	261	-454.0616772	0.12	0.192	-454.1229430	3.13	0.407
aσTZ0	705	552	-454.1097726	0.06	0.019	-453.4147975	1.24	0.066
aσQZ0	1158	996	-454.1229430	0.03	0.003	-453.5014976	0.54	0.013
	Guanine							
BS	#prim.	#cont.	RI-B3LYP+D3	er RI	%dis.Av.er.	RI-MP2	er RI	%dis.Av.er.
aDZ	440	298	-542.3591679	0.26	0.465	-541.175836	3.26	0.966
aTZ	763	621	-542.4846937	0.02	0.044	-541.627545	1.56	0.191
aQZ	1263	1110	-542.5225635	0.04	0.011	-541.777130	0.48	0.043
a5Z	1999	1797	-542.5333026	0.03		-541.829920	0.20	
aσDZ0	478	298	-542.4564502	0.19	0.205	-541.373927	3.86	0.433
aσTZ0	801	621	-542.5143007	0.06	0.022	-541.693092	1.55	0.076
aσQZ0	1301	1110	-542.5308608	0.03	0.004	-541.797126	0.64	0.009
	Cytosine							
BS	#prim.	#cont.	RI-B3LYP+D3	er RI	%dis.Av.er.	RI-MP2	er RI	%dis.Av.er.
aDZ	335	229	-394.7880656	0.18	0.530	-393.910167	2.25	1.003
aTZ	589	483	-394.8835741	0.02	0.049	-394.243722	1.14	0.190
aQZ	984	870	-394.9114710	0.03	0.012	-394.353978	0.35	0.048
a5Z	1567	1416	-394.9194149	0.02		-394.392803	0.15	
aσDZ0	364	229	-394.8628559	0.14	0.212	-394.058706	2.81	0.418
aσTZ0	618	483	-394.9055868	0.06	0.021	-394.292549	1.15	0.075
aσQZ0	1013	870	-394.9175567	0.02	0.006	-394.368880	0.49	0.028
	Uracil							
BS	#prim.	#cont.	RI-B3LYP+D3	er RI	%dis.Av.er.	RI-MP2	er RI	%dis.Av.er.
aDZ	324	220	-414.675660	0.21	0.514	-413.778141	2.33	1.003
aTZ	564	460	-414.776440	0.01	0.048	-414.125025	1.18	0.196
aQZ	936	824	-414.805970	0.03	0.010	-414.240598	0.35	0.045
a5Z	1484	1336	-414.814330	0.02		-414.281592	0.16	
aσDZ0	352	220	-414.756865	0.18	0.211	-413.9383023	2.88	0.436
aσTZ0	592	460	-414.800457	0.03	0.022	-414.1781659	1.12	0.074
aσQZ0	964	824	-414.812446	0.02	0.002	-414.2571118	0.48	0.007
